# Modulation of the Endothelin System in Colorectal Cancer Liver Metastasis: Influence of Epigenetic Mechanisms?

**DOI:** 10.3389/fphar.2020.00180

**Published:** 2020-03-03

**Authors:** Mohamed R. Mahdi, Rania B. Georges, Doaa M. Ali, Raouf F. Bedeer, Huda M. Eltahry, Abd-El Hakiem Z. Gabr, Martin R. Berger

**Affiliations:** ^1^ Toxicology and Chemotherapy Unit, German Cancer Research Center (DKFZ), Heidelberg, Germany; ^2^ Department of Human Anatomy & Embryology, Faculty of Medicine, Mansoura University, Mansoura, Egypt

**Keywords:** endothelin system, DNA methyltransferase, decitabine, epigenetic mechanism, colorectal cancer cell lines, liver metastasis

## Abstract

Targeting of endothelin system genes is a promising strategy in cancer therapy. The modulation of these genes was explored in a model of colorectal cancer (CRC) liver metastasis and in a panel of CRC tumor cell lines that were exposed to the demethylating agent decitabine. The CC531 rat model mimicking CRC liver metastasis was used for tumor cell re-isolation and analysis of the endothelin system genes and DNA methyltransferases (DNMTs) by microarray. To mimic the effects caused by methylation changes, a panel of seven CRC cell lines was treated with the demethylating agent decitabine. Three genes of the endothelin system were potently modulated at messenger RNA (mRNA) level in rat CC531 cells during liver colonization. The concomitant decrease of two DNMTs suggested an influence from altered methylation. Changes in gene expression were also accomplished by exposure of CRC cells to the demethylating agent decitabine, when using daily low concentrations for 3 days, with minimal cytotoxic effects. Sensitive human SW480 cells showed an almost 100fold upregulation of endothelin-1 mRNA compared to untreated cells. This, however, was different in LS174T cells, which showed no significant increase in gene expression although the methylation levels were significantly decreased at a variety of corresponding loci. We suggest that the mechanism induced by methylation on gene expression in metastatic CRC cells can be compromised. The results question the overall success of treating metastatic CRC by methylation inhibitors.

## Introduction

Colorectal cancer (CRC) is considered one of most common human cancers, as well as one of the common causes of cancer related death worldwide. Currently, CRC ranks at position four in cancer mortality and at position three in prevalence (Haggar and Boushey, 2009). The endothelin (EDN) system has an important role in a variety of physiological and pathophysiologic processes including pulmonary, cardiovascular, and renal diseases, wound healing, neurotransmission, and cancer biology (Khimji and Rockey, 2010). It comprises the three isoforms, endothelin-1 (EDN-1), endothelin-2 (EDN-2), endothelin-3 (EDN-3), the G-protein-coupled endothelin receptors type A (EDNRA) and B (EDNRB), as well as endothelin converting enzyme (ECE) (Barnes and Turner, 1997; Wang et al., 2013). All endothelins are characterized by a single α-helix with two disulfide bridges. Endothelin-1 acts through different signaling pathways and is involved in normal cell functions as well as those contributing to cancer development and progression, including cell proliferation, reduced apoptosis, epithelial mesenchymal transition, invasion, metastasis, and tumor angiogenesis. Based on these properties, EDN-1 and its signaling axis are considered very attractive targets for cancer therapy (Bagnato et al., 2011). EDN-2 is known as a hypoxia-inducible and anti-hypoxic factor. For their structural similarity, EDN-2 and EDN-3 can be considered as natural competitors for the EDN-1 receptor and as natural antagonists of EDN-1, and in line with this notion they have anticancer effects (Wang et al., 2013).

The receptors EDNRA and EDNRB are members of the class A of G protein-coupled receptors (GPCRs, the “druggable” class). The affinity of EDNRA to EDN-1 and EDN-2 is twofold higher than its affinity to EDN-3, while the affinity of EDNRB is equal to EDN-1, EDN-2, and EDN-3 (Rosano et al., 2013). EDNRA has an unequal distribution in the colon, as it is over-expressed in the proximal and distal segments of the colon (Hoosein et al., 2007). EDNRB is considered as the predominant receptor in normal colon, but in colon cancer it is down-regulated in cancer associated blood vessels, fibroblasts, and epithelial cells (Hoosein et al., 2007). In colon cancer, EDNRA expression is more dominant than EDNRB (Irani et al., 2014). Epigenetic alteration of EDNRB expression by aberrant methylation has an important role in the pathogenesis of hepatocellular carcinoma (Hsu et al., 2006), and in gastric cancer (Tao et al., 2012). Endothelin converting enzyme 1 (ECE1) and endothelin converting enzyme 2 (ECE2) are membrane-bound, zinc-binding metalloproteases (Mckenzie et al., 2014).

Decitabine (DAC; 5-aza-2'-deoxycytidine) is a deoxycytidine analog (trade name Dacogen), which contains nitrogen instead of a carbon in the five-position of cytosine (Momparler, 2005b). After entering the intracellular space, DAC is activated and converted by phosphorylation to its triphosphate form. After its incorporation into DNA, the nucleoside analogue blocks DNA methyltransferases and causes passive demethylation of DNA (Karahoca and Momparler, 2013). DAC inhibits DNA methyltransferases; DNMT1, DNMT3a, and DNMT3b irreversibly by forming covalent bonds with the active sites of DNA methyltransferase (Christman, 2002; Oka et al., 2005). DAC reactivates tumor suppressor genes, stimulates differentiation or senescence, and inhibits tumor growth and clonogenic ability. In animal models, DAC has antitumor effects against leukemia and solid tumors (Momparler, 2005a). Pharmacokinetic studies showed that the DAC effect is related not only to the dose, but also the duration of exposure (Momparler, 2005b).

Hypomethylating agents (HMAs) were approved for treatment of hematologic malignancies, but with little success in solid tumors (Azad et al., 2017; Lee et al., 2018). EGFR promoter hypermethylation in colorectal cancer has been found associated with patient resistance to anti-EGFR therapies (Jueliger et al., 2016). In line with this, a combination therapy with DAC and panitumumab showed a promising effect in patients with wild-type KRAS metastatic colorectal cancer (Garrido-Laguna et al., 2013). Accordingly, low doses of DAC were beneficial in advanced hepatocellular carcinoma patients (Mei et al., 2015). In ovarian cancer, genes encoding epigenetic regulators were found mutated (Moufarrij et al., 2019). Because epigenetic modifiers as DAC have emerged as potential cancer therapeutics, DAC therapy can sensitize malignancies to platinum therapy in ovarian cancer patients (Matei et al., 2012).

The role of EDN-1 in cancer may be due to over-expression of EDN-1 or overexpression of its receptors, or their signaling pathways. These changes in the EDN-1 signaling axis are due to genetic alterations and/or epigenetic changes, as DNA methylation and histone modification (Rosano et al., 2013). In a recent study, low to undetectable expression levels of EDN-2 and EDN-3 were found in human and rat colon cancers, but normal levels in matched control tissues. The reduced expression of EDN-2 and EDN-3 resulted from epigenetic silencing. In a rat model of colonic tumorigenesis, EDN-2 and EDN-3 expression was lost several weeks before the onset of colon cancer. However, if these rats were treated with 5-aza-2′-deoxycytidine, their colonic mucosa showed re-expression of both, EDN-2 and EDN-3, but with a preference for EDN-2. Moreover, forced re-expression of EDN-2 and EDN-3 in human colon cancer cells led to inhibition of invasion and migration *in vitro*. Thus, in human and rat colon cancer cell lines as well as in human primary colon cancers, there is down-regulation and hyper-methylation of the EDN-2 and EDN-3 genes (Wang et al., 2013).

In this study, we first studied a rat model for colorectal cancer liver metastasis and followed the changes in gene expression from re-isolated CC531 rat colorectal cancer cells. Remarkably, the endothelin system showed early and distinct modulation of gene expression in response to progressing liver colonization by these cells. We then hypothesized that these changes are caused by epigenetic alterations, mainly by changes in methylation. To that purpose, rat and human CRC cells were exposed to the demethylating agent DAC and followed for expression changes of the endothelin system. This study aimed toward understanding the mechanism of the gene modulation observed during liver colonization and exploring the value of DAC in modulating the endothelin system.

## Materials and Methods

### Colon Adenocarcinoma Cell Lines

Six human (LS174T, SW480, SW620, HT29, CACO2, SW707) and one rat (CC531) colorectal adenocarcinoma cancer cell lines were obtained and cultured routinely in RPMI-1640 media (Invitrogen), supplemented with 10% fetal calf serum (FCS), penicillin and streptomycin (100 IU/ml, 100 μg/ml), and L-glutamine (2 mM) (Invitrogen). Cancer cells were kept under standard cell culture conditions (at 37°C, in humidified air with 5% CO_2_) and passaged every 2 to 3 days to keep them in logarithmic growth. For isolation and propagation, cells were first washed with phosphate-buffered saline (PBS), then trypsinized (0.25% trypsin), pelleted (at 1,500 rpm, 5 min), and finally suspended at the desired concentration in fresh medium.

### Decitabine Handling and Concentration Selection

DAC was obtained from Pfizer Comp. (Illertissen, Germany). A fresh 10 mM stock solution of DAC was dissolved in phosphate-buffered saline (PBS), respectively, for each experiment. Working solutions were prepared by serial dilution and used to expose the cells to final concentrations of 0.25 to 32 µM. After determining the cytotoxic effect of DAC, we assessed in all CRC cell lines the range of DAC concentrations that were tolerated with no to minimal cytotoxic effects. These concentrations were administered daily for different periods to modulate the gene expression of the endothelin system.

### RNA Isolation and Complementary DNA Synthesis

RNeasy Mini Kit spin technology (QIAGEN^®^) was used for total RNA isolation following the manufacturer's protocol, then the concentration and purity of RNA samples was measured by a NanoDrop spectrophotometer (NanoDrop Technologies, Wilmington, DE). Afterwards, complementary DNA (cDNA) was synthesized by Maxima reverse transcriptase enzyme (Thermo Scientific, Schwerte, Germany).

### Real-Time PCR and Gel Electrophoresis

Red Taq DNA polymerase (Genaxxon) was used for amplification of endothelin system gene templates from cDNA samples (2 µl each) in a Mastercycler^®^ nexus gradient (Eppendorf). For protocol and primers see **Supplementary Materials**. After that, the DNA templates were electrophoresed on a 2% precast agarose gel. The gels were then stained with ethidium bromide, visualized by UV light and photographed by a Bio-Rad gel station (Munich, Germany).

### Quantitative Real-Time PCR

Real-time PCR was done using 2× LightCycler 480 Master Mix along with the appropriate probes from the Human Universal Probe library (Roche, Mannheim, Germany). Three replicates were amplified for each sample, and the expression level of a reference gene [glyceraldehyde 3-phosphate dehydrogenase (GAPDH)] was used for normalization of expression data. The data were calculated with the 2^−ΔΔCT^ method and expressed as fold changes (Livak and Schmittgen, 2001). Detailed information about the protocols used and the primer sequences are given in the **Supplementary Materials**.

### Preparation of Tumor Cells for Injection

The stably transfected LS174T^luc^ cells and CC531^luc/RFP^ cells were washed with PBS, trypsinized, pelleted, and suspended at a concentration of 4×10^6^ cells/500 μl (350 μl PBS+150 μl Biomatrix EHC; Serva Electrophoresis, Heidelberg, Germany).

### Tumor Cell Injection and Re-Isolation

CC531^luc/RFP^ cells were injected into 6 to 8-week-old male WAG/Rij rats (Charles River Laboratories, Germany). Tumor cell re-isolation was performed as described before (Georges et al., 2012). Briefly, CC531 cells were intraportally implanted into the liver of Wag-Rij rats and re-isolated at various time intervals (after 3, 6, 9, 14, and 21 days) following their intraportal administration.

LS174T^luc^ cells were injected into RNU nude male rats (Charles River, Sulzfeld, Germany) at a corresponding body weight of 90–120 g. The rats were left for 1 week adaptation period, and then were operated to inject 5 × 10^6^ LS174T cells *via* a mesocolic vein. The cells migrate to the liver through the portal vein and start to form liver nodules. After 4 weeks, the rats were sacrificed to re-isolate tumor cells for RNA extraction and microarray analysis. The detailed protocols for operation were described previously (Eyol et al., 2012).

Rats were fed a standard diet *ad libitum* and given an adaptation period of 1 week prior to any experimental procedures. All animal experiments were approved by the responsible governmental animal ethics committee (RP Karlsruhe, Germany).

### Microarray Analysis

Three sets of RNA microarray analyses were performed. The first microarray was used to analyze the alterations in the expression profile of ca. 22,500 rat genes for discovering a possible correlation with liver metastasis formation of CC531 cells. To that purpose, CC531 cells were intraportally injected into Wag-Rij rats and re-isolated from rat livers at several time points (3, 6, 9, 14, and 21 days) after tumor cell inoculation (Georges et al., 2012).

The second microarray was used to determine, which endothelin genes were affected by DAC treatment *in vitro*. LS174T and SW620 human CRC cell lines were treated with different DAC concentrations, ranging from 0.25 to 2 µM. The DAC exposure was repeated, respectively, daily for 3 days. Then the gene expression was analyzed in treated and control groups.

The third microarray was performed to compare expression between *in vitro* and *in vivo* conditions. LS174T cells were treated with three daily doses of 0.25 µM DAC, and then the cells were collected and injected in the mesocolic vein of nude rats to mimic the liver metastasis process. After 3 weeks *in vivo*, cells were re-isolated from rat liver and cultivated in puromycin-containing media for 6 days *in vitro* before collection of total RNA for microarray. Then, the gene expression levels were compared between cells treated either *in vivo* or *in vitro*.

For RNA isolation from tumor cells, the RNeasy mini-kit (Qiagen, Hilden, Germany) was used. RNA was eluted in water. The quality of total RNA was checked by gel analysis and the total RNA nanochip assay on an Agilent 2100 Bio-analyzer (Agilent Technologies GmbH, Berlin, Germany). Only samples with RNA index values >8.5 were selected for expression profiling. RNA concentrations were determined using a NanoDrop spectrophotometer (NanoDrop Technologies, Wilmington, DE). Microarray probe labeling and Illumina Sentrix BeadChip array hybridization were performed as described before (Horrix et al., 2011; Georges et al., 2012). Biotin-labeled complementary RNA (cRNA) samples for hybridization on Illumina Rat Sentrix-12 BeadChip arrays for rat CC531 cells and on illumina_humanht-12 _v4 for human LS174T & SW620 cells (Illumina, San Diego, CA) were prepared according to Illumina's sample labeling procedure based on the modified Eberwine protocol.

#### Infinium Methylation Assay

LS174T human CRC cells were used for methylation analysis in response to DAC. Cells were exposed to 0.25 µM DAC for 72 h. Then, the cells were harvested and DNA was extracted by the QIAamp DNA Mini Kit (QIAGEN^®^) according to the manufacture's protocol. DNA concentrations were determined using a NanoDrop spectrophotometer (NanoDrop Technologies, Wilmington, DE).

Genome-wide screening of DNA methylation patterns was performed by using the Infinium MethylationEPIC BeadChips (Illumina, San Diego, US), allowing the simultaneous quantitative measurement of the methylation status at 865,918 CpG sites. By combining Infinium I and Infinium II assay chemistry technologies, the BeadChip provides coverage of 99% of RefSeq genes and 96% of CpG islands.

DNA concentrations were determined using PicoGreen (Molecular Probes Inc., Eugene, USA). The quality of genomic DNA samples was checked by agarose-gel analysis, and samples with an average fragment size >3 kb were selected for methylation analysis. The laboratory work was done in the Genomics and Proteomics Core Facility at the German Cancer Research Center, Heidelberg, Germany (DKFZ). (For more detail please refer to **Supplementary Materials**).

### Western-Blotting

Colorectal cancer cells were collected; and proteins from fresh cell lysates were separated by electrophoresis on prefabricated gels (Serva) at 150 V for 90 min in 1 X Laemmli buffer. The blotting of separated proteins was done onto a polyvinylidene fluoride (PVDF) membrane. For specific band detection, these antibodies were used; endothelin1 primary antibody was used (ET-1 (N-8): sc-21625, Santa Cruz) as well as β-actin as a loading control (Actin (C-11): sc-1615, Santa Cruz), and donkey anti-goat antibody was used as secondary antibody (sc 2020, Santa Cruz). Levels of β-actin were used to normalize the protein expression. A detailed description of the respective protocol is given in the **Supplementary Materials**.

### 3-(4.5-Dimethylthiazol-2-yl)-2.5-Diphenyl Tetrazolium Bromide Cell Viability Assay

CRC cells were transferred at pre-optimized cell density into 96 well-plates (flat bottom, Becton-Dickinson, Heidelberg, Germany) (100 μl medium/well). To measure proliferation rates, cells were exposed to increasing concentrations of DAC for four time periods (24, 48, 72, and 96 h). Surviving cell fractions (eight replicates/sample) were calculated in the treated and control groups. 10 μl MTT [3-(4.5-dimethylthiazol-2-yl)-2.5-diphenyl tetrazolium bromide, Serva Electrophoresis GmbH, Heidelberg, Germany] were added and incubated with cells for 3 h at 37°C. Then, an equal volume of acidified isopropanol (0.04 mM HCl-isopropanol) was added. The absorption from the dissolved formazan crystals was measured at 540 nm (reference filter of 690 nm) in an automated microtiter plate spectrophotometer (anthos Mikrosysteme GmbH, Krefeld, Germany). CRC cell survival rates with DAC were expressed as percentage of control, and GraphPad Prism 6 software was used to calculate the inhibitory concentrations (IC50).

### Statistical Analysis

For calculating the gene expression levels in RT-PCR, the samples were amplified in triplicate and intensities of the target genes were normalized to the reference genes (GAPDH or γ-tubulin) by the ΔΔCT method (Livak and Schmittgen, 2001). Student's t-test was used to determine the significance of results. GraphPad Prism 6 software was used for graphical presentation and statistical significance. P values ≤0.05 were considered statistically significant.

## Results

### Modulation of the Endothelin System Members in CC531 Cells During Liver Colonization

**Figure 1:**
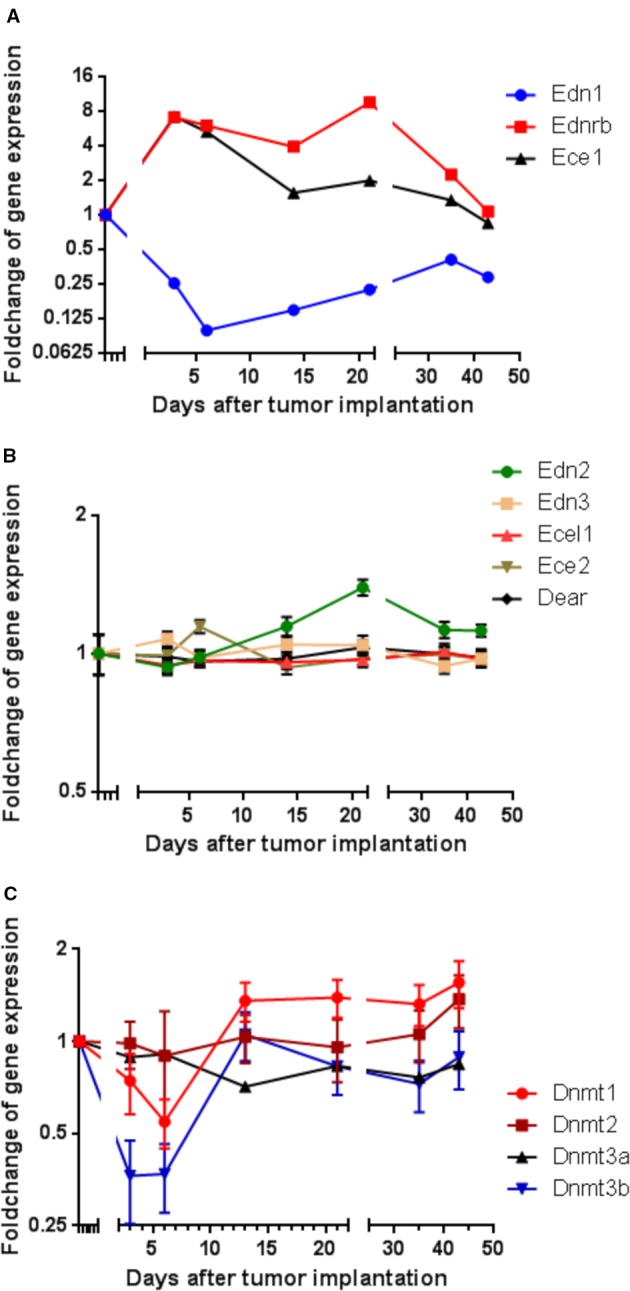
Modulation of gene expression in rat CC531 colorectal cancer cells, which were re-isolated after different periods of liver colonization, as shown by microarray analysis. **(A)** Expression profile of endothelin 1 (*Edn1)*, endothelin-converting enzyme 1 (*Ece1)*, and endothelin receptor B (*Ednrb)* in re-isolated CC531 cells. **(B)** Expression profile of endothelin 2 (*Edn2)*, endothelin 3 (*Edn3)*, endothelin-converting enzyme 2 (*Ece2)*, endothelin-converting enzyme-like 1 (*Ecel1),* and dual endothelin-1/angiotensin II receptor (*Dear)* in re-isolated CC531 cells. **(C)** Expression profile of DNA methyltransferase 1 (*Dnmt1)*, DNA methyltransferase 2 (*Dnmt2)*, DNA methyltransferase 3a (*Dnmt3a),* and DNA methyltransferase 3b (*Dnmt3b)* in re-isolated CC531 cells. The values represent the fold change in gene expression as determined by microarray in re-isolated CC531 cells that were colonizing the rat liver in comparison to the expression in cells growing *in vitro*. The x-axis gives the time (in days) after intraportal implantation of CC531 cells to syngeneic rats. Each point corresponds to one time point, after which the cells were re-isolated. The y-axis gives the messenger RNA (mRNA) expression of the studied gene relative to that level found in CC531 cells growing *in vitro.*

To identify genes that are involved in the metastatic behavior of CRC cells, cDNA microarray technique was used to monitor messenger RNA (mRNA) expression in rat CC531 cells for changes related to their homing into the liver. CC531 cells were intraportally injected into rat livers and re-isolated after various time intervals following their intraportal administration for mRNA expression analysis. Regarding the endothelin system, there was a distinct mRNA expression modulation of *Edn1*, *Ednrb*, and *Ece1* in CC531 cells colonizing rat liver (**Figure 1A**).

For endothelin receptor B (*Ednrb*) and endothelin converting enzyme 1 (*Ece1*; **Figure 1A**), a 7–9fold increase in mRNA expression was observed during the initial phase of liver colonization. In addition, endothelin 1 (*Edn1*) was maximally 10fold downregulated, but showed a short delay compared to *Ednrb* and *Ece1*. Common to all three genes was a gradual return to an expression ratio, which was similar to that of control cells growing *in vitro* after the respective tumor cells had been re-isolated and were grown under standard culture conditions (37°C, humidified atmosphere with 5% CO_2_). In variance, the other endothelin system members [endothelin 2 and 3 (*Edn2*, *Edn3*), endothelin converting enzyme like 1 (*Ecel1*), endothelin converting enzyme 2 (*Ece2*), and dual endothelin 1/angiotensin II receptor (*Dear*)] showed no significant modulation of RNA expression (**Figure 1B**). These alterations raised the question, whether the changes observed in endothelin system members' expression were caused by epigenetic changes and whether changes in methylation could explain the observed effects. In fact, there was an initially decreased expression of the DNA methylating enzymes *Dnmt1* and *Dnmt3b* by 45 and 73% in the same cells (**Figure 1C**), which might be responsible for reduced cellular methylation activity and consequently increased expression of genes.

To mimic the reduced *Edn1* expression, a knockdown strategy was initially followed. However, neither rat nor human small interfering RNAs (siRNAs) directed against endothelin 1 (for sequences and experimental details see **Supplementary Materials**) were successful in achieving a significant knockdown (data not shown).

### Effects of Decitabine Treatment on Different Colorectal Cancer Cell Lines

To investigate possible expression changes in endothelin system members due to altered methylation, the hypomethylating agent (HMA) DAC was used in a panel of CRC cell lines, consisting of rat CC531 cells and up to six human cell lines. As the cytotoxic effect of HMAs can vary between cancer cell lines and depends on concentration and exposure time used, the next experiment concentrated on the optimal dose and exposure time of DAC.

**Figure 2:**
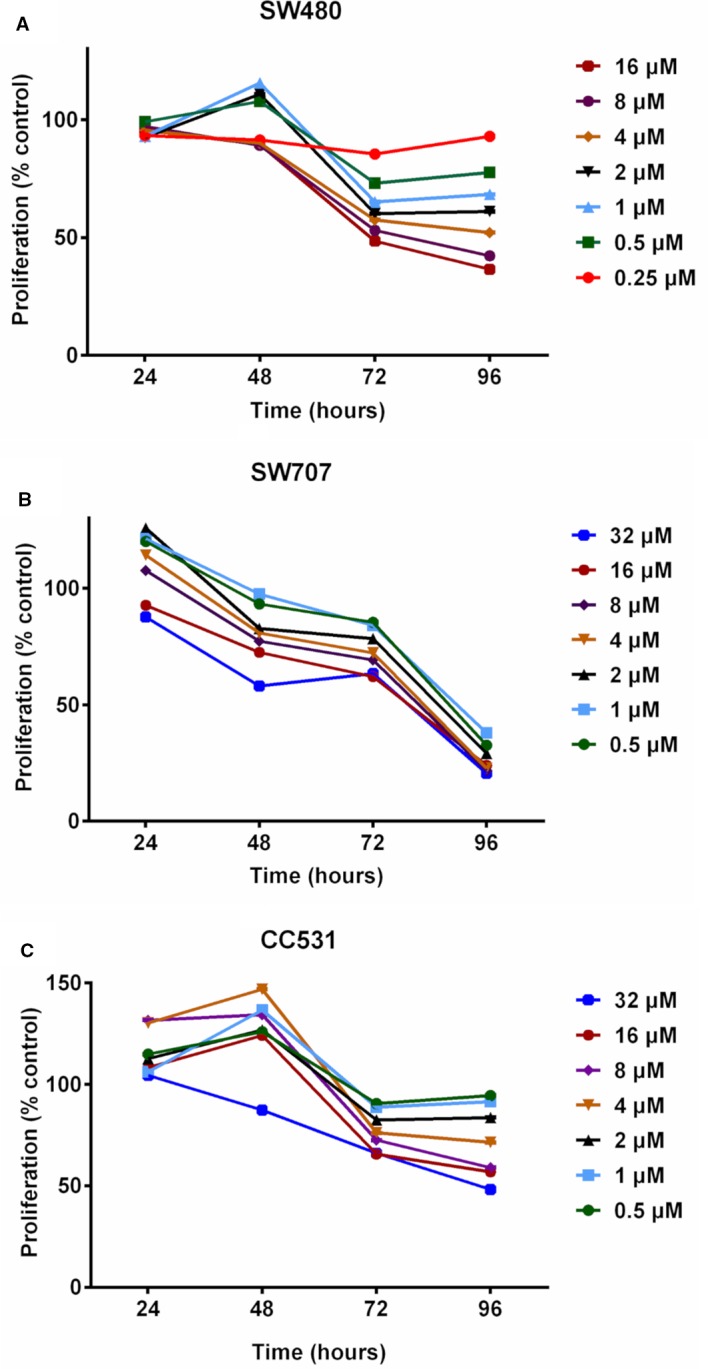
Effects of exposing three colorectal cancer cell lines to increasing concentrations of decitabine (DAC) on their proliferation, as determined by MTT assay. **(A)** Proliferation of human SW480 cells in percent of untreated control after exposure to increasing concentrations (0.25, 0.5, 1, 2, 4, 8, 16 µM) of decitabine (DAC) for 24 to 96 h. **(B)** Proliferation of human SW707 cells in percent of untreated control after exposure to increasing concentrations (0.5, 1, 2, 4, 8, 16, 32 µM) of decitabine (DAC) for 24 to 96 h. **(C)** Proliferation of rat CC531 cells in percent of untreated control after exposure to increasing concentrations (0.5, 1, 2, 4, 8, 16, 32 µM) of decitabine (DAC) for 24 to 96 h.

We first evaluated daily exposure to DAC (0.25–32 µM concentrations), which decreased the proliferation of CRC cells in a concentration-dependent manner (**Figure 2**). Human SW480 cells (**Figure 2A**) showed mildly increased proliferation at 48 h in response to intermediate concentrations of DAC (0.5–2 µM), but at 96 h there was a concentration-dependent effect on cell survival ranging in fraction from 38% (DAC;16 µM) to 95% (DAC; 0.25 µM).

Human SW707 cells were more sensitive to DAC than SW480 cells. After an initial mild stimulation, the ratio of surviving cells ranged from 38% (0.5 µM) to 24% (32 µM) after 96 h of exposure to DAC (**Figure 2B**).

Rat CC531 cells were less sensitive to DAC than the two human CRC cell lines SW480 and SW707. In the rat cells, daily DAC exposure caused initial stimulation of proliferation, followed by proliferation inhibition. Interestingly, 0.5 and 32 µM concentrations for 96 h caused proliferation inhibition of less than 10% and slightly more than 50%, respectively (**Figure 2C**).

**Figure 3:**
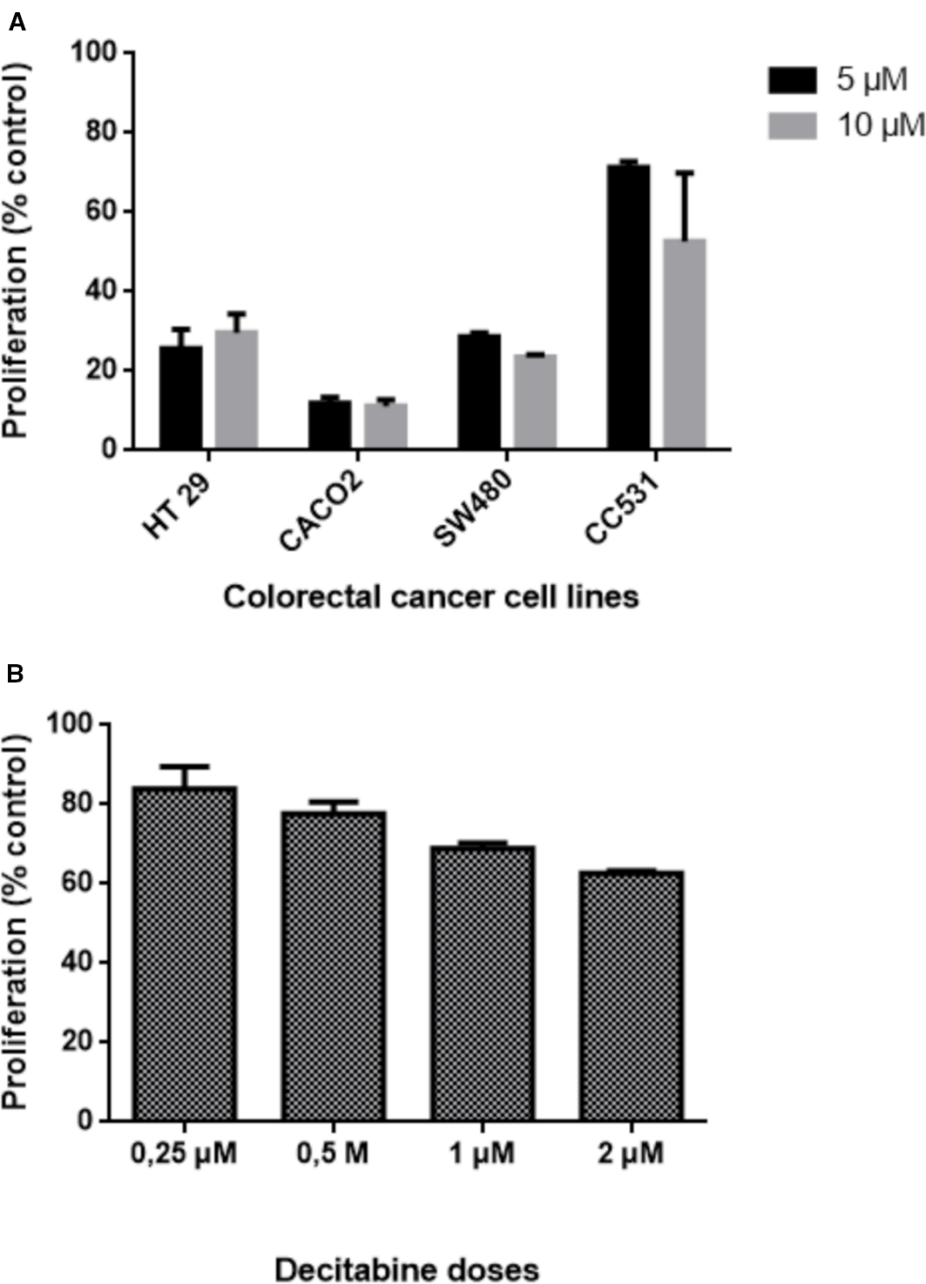
Effects of exposing five colorectal cancer cell lines to different concentrations of decitabine (DAC) on their proliferation, as determined by 3-(4.5-dimethylthiazol-2-yl)-2.5-diphenyl tetrazolium bromide (MTT) assay. **(A)** Proliferation of human HT29, CACO2, SW480, and rat CC531 cells after exposure to two high concentrations (5 and 10 µM) of decitabine (DAC), administered daily for 3 days. **(B)** Proliferation of human LS174T cells after exposure to four low concentrations (0.25, 0.5, 1.0, 2.0 µM) of decitabine (DAC), administered daily for 3 days.

The difference in sensitivity to DAC was evident also for the other human cell lines: HT29, Caco2, and SW480 cells were all more sensitive to DAC exposure than rat CC531 cells (**Figure 3A**). Compared to these cell lines, LS174T was in the same range of sensitivity to DAC, as concentrations below 1 µM were well tolerated (**Figure 3B**).

**Figure 4:**
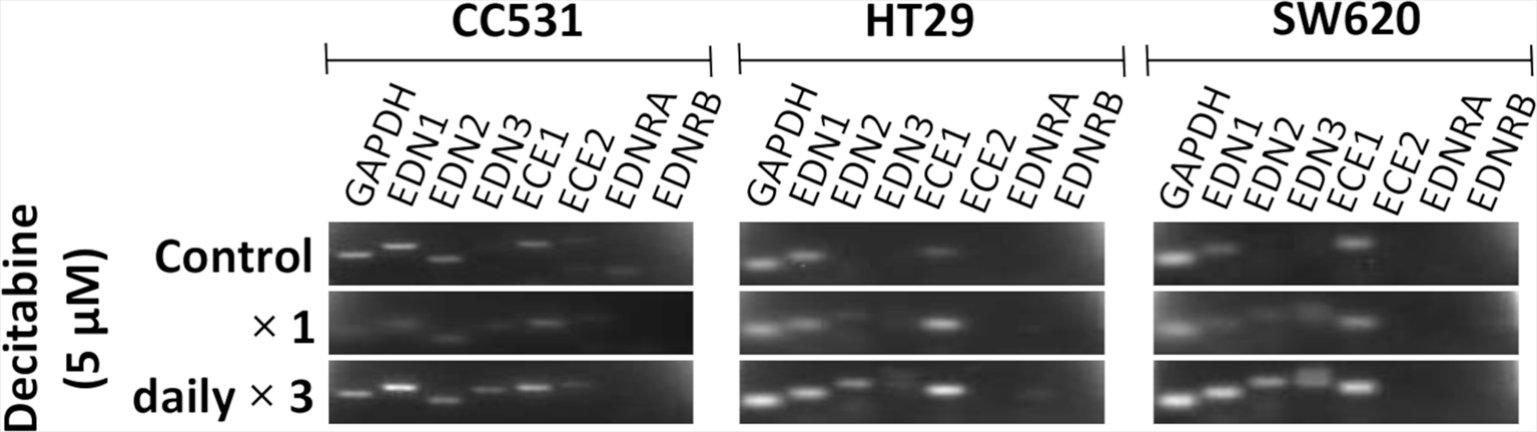
Messenger RNA (mRNA) expression of endothelin system members endothelin-1 (*EDN1)*, endothelin-2 (*EDN2)*, endothelin-3 (*EDN3)*, endothelin-converting enzyme 1 (*ECE1)*, endothelin-converting enzyme 2 (*ECE2)*, endothelin receptor A (*EDNRA)*, and endothelin receptor (*EDNRB*) in two human (HT29 and SW620) and in rat CC531 colorectal cancer cells after exposure to decitabine (DAC, 5 µM) either as a single administration (second row) or as a daily administration over 3 days (third row) in comparison to untreated control cells (first row) as detected by real-time (RT)-PCR. The housekeeping gene glyceraldehyde 3-phosphate dehydrogenase (GAPDH) was used as loading control.

Inhibition of DNMT by DAC leads to changes in the expression level of certain genes. In an initial approach to assess the influence of DAC on the expression of endothelin system members, CC531 cells were compared with HT29 and SW620 cells. Single administration of DAC (5 µM) did not alter the expression of endothelin system members. However, exposure to this concentration daily for 3 days caused increased expression of *Edn3* and *Ece2* in CC531 cells, and of *EDN2*, *EDN3*, and *ECE1* in HT29 and SW620 cells (**Figure 4**).

**Figure 5:**
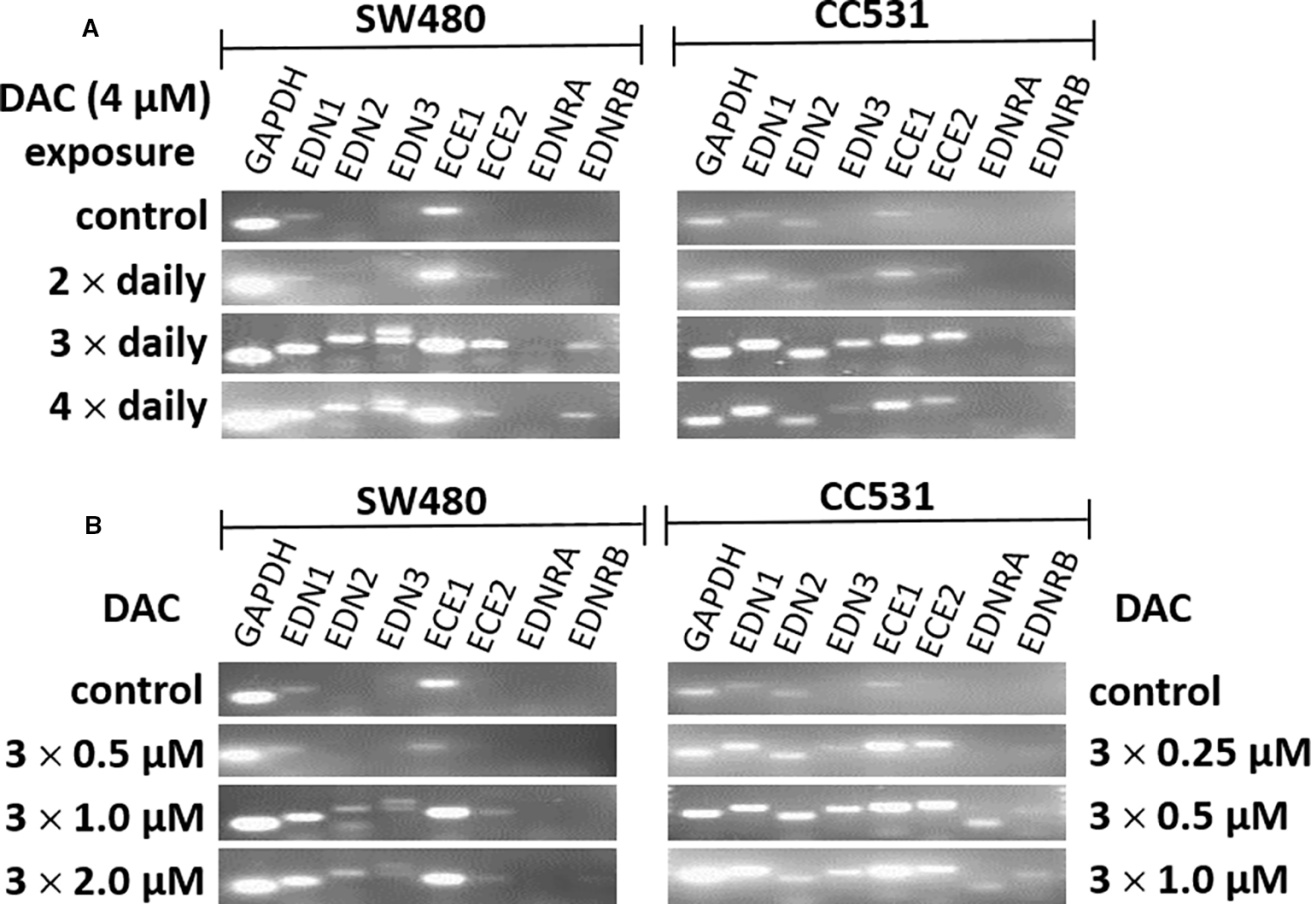
mRNA expression of endothelin system members (*EDN1*, *EDN2*, *EDN3*, *ECE1*, *ECE2*, *EDNRA*, and *EDNRB*) as detected by real-time (RT)-PCR in human (SW480) and rat (CC531) colorectal cancer cells. Glyceraldehyde 3-phosphate dehydrogenase (GAPDH) was used as housekeeping gene. **(A)** Decitabine (DAC) at a constant concentration (4 µM), but varied exposure time [two (second row), or three (third row), or four (fourth row) daily administrations]. **(B)** Decitabine (DAC) with three increasing concentrations but constant exposure period (0.5, 1.0, 2.0 µM in SW480 and 0.25, 0.5, 1.0 µM in CC531 cells; administered once daily for 3 days).

Further optimization of the DAC effect on gene expression was done in CC531 and SW480 cells. Exposure to reduced (4 µM) daily concentrations of DAC showed that an optimal effect was reached after 3 days, which could not be further improved by exposure to 4 days (**Figure 5A**). Under these conditions, all three endothelins, their converting enzymes, and endothelin receptor B were increased in expression in CC531 and SW480 cells. Quantitative RT-PCR showed that *EDN1* mRNA increased by up to 93fold in SW480 cells (**Table 1**).

**Table 1:** Expression of *EDN1* mRNA in SW480 cells after daily exposure to decitabine as shown by real-time (RT)-PCR.

DAC concentration	ΔC_T_ ^a)^	ΔΔC_T_ ^b)^	Fold change^c)^
2 × daily (4 µM)	13.59	−2.93	7.64
3 × daily (4 µM)	11.32	−6.54	93.27
4 × daily (4 µM)	10.85	−5.68	51.15
3 × daily (0.25 µM)	16.02	−1.84	3.57
3 × daily (0.5 µM)	14.75	−3.11	8.61
3 × daily (1 µM)	12.85	−5.01	32.30
3 × daily (2 µM)	11.6	−6.26	76.64

a) ΔC_T_= C_T_ (EDN1)-C_T_ (GAPDH) (C_T_; threshold cycle: the PCR cycle at which the fluorescent signal of the reporter dye crosses an arbitrarily placed threshold).

b) ΔΔC_T_= ΔC_T_ (treated sample)-ΔC_T_ (untreated sample).

c) Fold change= 2^−(∆∆Ct)^.

Reductions of this daily dose to 0.5–2.0 µM (SW480) or 0.25–1 µM (CC531) were less active in terms of gene expression. Quantitative RT-PCR showed that the 2 µM concentration in SW480 cells did not reach that level observed in response to 4 µM, and that the lowest concentration used (0.25 µM) was barely active, at all (**Table 1**). However, exposure of CC531 cells to 0.5 and 1.0 µM concentrations of DAC induced expression of *EdnrA* and *EdnrB* (**Figure 5B**).

In an attempt to explore the effects of DAC exposure on the methylation profile of endothelin system members, LS174T human CRC cells were exposed to 0.25 µM DAC for 72 h, harvested, and their DNA was extracted for methylation analysis.

**Figure 6:**
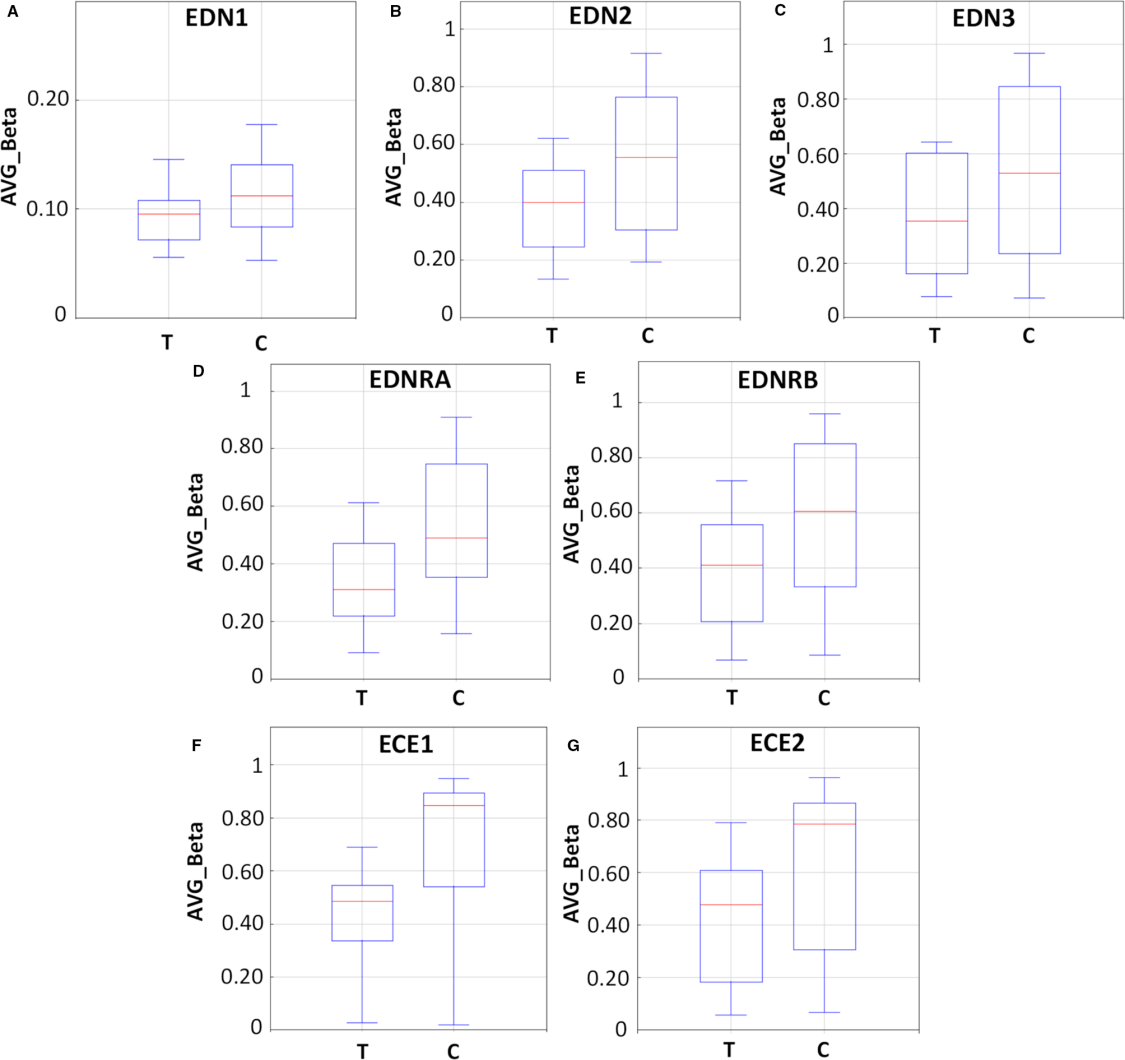
Methylation analysis of the endothelin system members (EDN1, EDN2, EDN3, EDNRA, EDNRB, ECE1, and ECE2) in LS174T human colorectal cancer (CRC) cells after exposure to decitabine (DAC, 0.25 µM), administered once daily for 72 h. Box plots show the average beta (AVG_Beta) methylation levels of gene loci that were differentially methylated in the genome of DAC-treated (T) and untreated control (C) LS174T cells. An average β-value (AVG_Beta) for each CpG locus ranging from 0 (unmethylated) to 1 (completely methylated) was extracted utilizing the GenomeStudio program (Illumina) for DNA extracted from control (C) and treated (T) LS174 cells. **(A**–**C)** Boxplots show the AVG_Beta methylation levels of 12, 13, and 24 CpG loci in the EDN1, EDN2, and EDN3 genes, respectively, in treated and control LS174T cells. **(D**, **E)** Boxplots show the AVG_Beta methylation levels of 30 and 63 CpG loci in the EDNRA and EDNRB genes, respectively in treated and control LS174T cells. **(F**, **G)** Boxplots show the AVG_Beta methylation levels of 121 and 38 CpG loci in the ECE1 and ECE2 genes, respectively in treated and control LS174T cells.

As demonstrated in **Figure 6** and **Table S4**, endothelin system genes in DAC-treated (T) LS174T cells showed a hypo-methylation pattern as compared to their untreated (C) counterparts. The boxplots (**Figures 6A–C**) show reduced methylation levels (AVG-Beta) of 12, 13, and 24 CpG loci in the *EDN1*, *EDN2*, and *EDN3* genes, respectively. In addition, 30 and 63 CpG loci in the *EDNRA* and *EDNRB* genes were hypo-methylated (boxplots **Figures 6D, E**) in response to treatment. In the endothelin converting enzyme genes *ECE1* and *ECE2,* a total of 121 and 38 CpG loci showed hypo-methylation in treated cells (boxplots **Figures 6F, G**).

**Figure 7:**
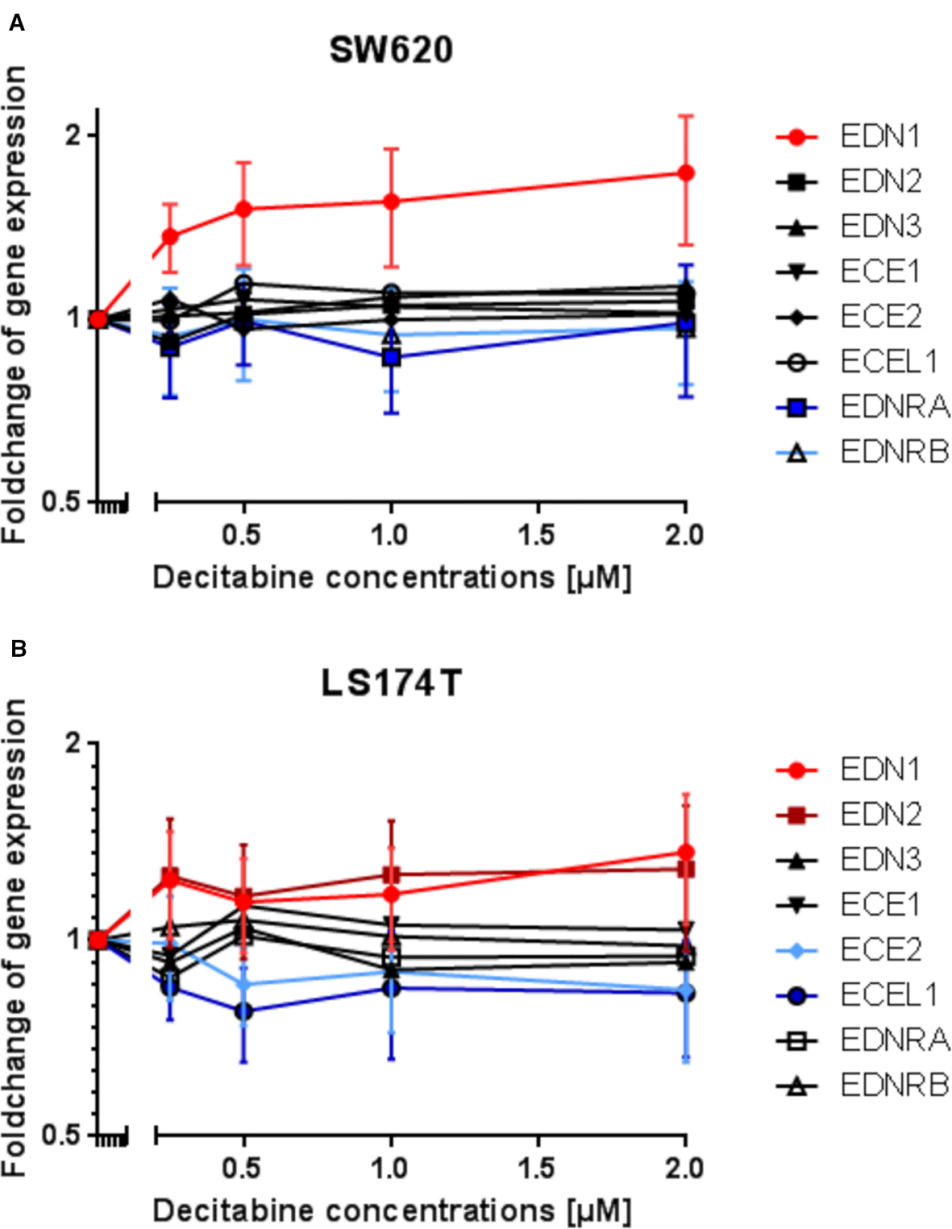
Modulation of endothelin system members (*EDN1*, *EDN2*, *EDN3*, *ECE1*, *ECE2*, *ECEL1*, *EDNRA*, and *EDNRB*) after exposure to three daily doses of incrementing decitabine (DAC) concentrations (0.5, 1.0, 1.5, 2.0 µM) in two human (SW620 and LS174T) colorectal cancer cell lines as observed by microarray analysis. **(A)** Fold-change of gene expression of endothelin system members in SW620 cells. **(B)** Fold-change of gene expression of endothelin system members in LS174T cells. The values represent the fold change in gene expression as determined by microarray in CRC cells that were exposed to DAC. The x-axis gives the decitabine (DAC) concentration (µM). The y-axis gives the messenger RNA (mRNA) expression of the studied gene relative to that level found in control cells.

Subsequently, a gene expression analysis was performed by microarray in DAC-exposed SW620 and LS174T colorectal cancer cells. It was expected, that the genes' expression would reflect the results observed from the methylation analysis. However, with the exception of *EDN1* in SW620 cells and possibly of *EDN1* and *EDN2* in LS174T cells, which showed increased expression, all other genes showed no modified or even decreased expression in response to DAC (**Figures 7A, B**).

In addition, a comparison of LS174T cells, which had been exposed to 0.25 µM DAC *in vitro* with corresponding LS174T cells that had been first exposed to DAC (0.25 µM), then grown in rats for 3 weeks before re-isolation and re-cultivation for another 6 days, showed no significant difference in the expression of endothelin system members (data not shown).

When analyzing the microarray data for concomitantly changed expression levels of DNMTs, there were no significant modulations, except for *DNMT1* in LS174T cells, which showed a concentration-dependent decreased expression, with a maximum of −34% (**Supplementary Material: Figure S1**).

## Discussion

Liver metastasis by gastrointestinal tract cancers is a complex process, which involves several sub-processes, including but not limited to cancer cell detachment, lysis of the extracellular matrix, invasion and endothelial transmigration, survival of tumor cells in the circulation, extravasation, and organ colonization (Massague and Obenauf, 2016). We have tried to analyze the process of liver colonization through gastrointestinal tumor cells by using animal models, characterized by a defined onset (i.e., tumor cell implantation into a mesocolic vein), after which these tumor cells were re-isolated following defined time intervals for subsequent analysis of changes in gene expression (Georges et al., 2011; Georges et al., 2012; Al-Taee et al., 2014; Sagini et al., 2018).

When analyzing the gene expression modulation during liver colonization by CC531 cells, an unexpected observation was made: These cells showed an 8-fold upregulation of *EndrB* and *Ece1* within 3 days after their implantation into a mesocolic vein. This increase was mirrored by a decrease in *End1*, which occurred with a minimal delay, as if the *Edn1* decrease were a reaction to the increase in *EdnrB* and/or *Ece1* mRNA levels. The other members of the endothelin system did not show a significant modulation of their mRNA expression.

At first sight, this scenario was not in line with the literature notion, according to which silencing of *EdnrB* by aberrant methylation has an important role in the pathogenesis of hepatocellular carcinoma (Hsu et al., 2006), and in gastric cancer (Tao et al., 2012). Similarly, it contradicts the notion that *EDN1* is a proper target when treating progressing cancers (Rosano et al., 2013). Therefore, experiments were set up, which aimed at elucidating the role of this modulation in CRC cells.

The endothelin system consists of endothelin-1 (EDN-1), endothelin-2 (EDN-2), endothelin-3 (EDN-3), the G-protein-coupled receptors, endothelin receptor type A (EDNRA) and endothelin receptor type B (EDNRB), endothelin converting enzyme 1 (ECE1), and endothelin converting enzyme 2 (ECE2) (Wang et al., 2013; Mahdi et al., 2014). Endothelin-1 has an enhancing effect on cancer cell proliferation, survival, invasion, and migration; it also has an effect on cancer stroma cells such as fibroblasts [proliferation, migration, contraction, extracellular matrix (ECM) remodeling], macrophages (chemo-attraction, ECM remodeling), and endothelial cells (proliferation, angiogenesis) (Bhalla et al., 2009). It has been reported that prepro-EDN-1, the pre-protein of endothelin-1, was overexpressed in human colon adenomas and adenocarcinomas as compared to normal colon (Egidy et al., 2000; Khimji and Rockey, 2010). Thus, beside their potential role for targeted cancer therapy, endothelin system members could also be used as early biomarker of cancer, as there are e.g., high plasma levels of Big-EDN-1 in patients with CRC. Similarly, loss of EDN2 or EDN3 in rat colon mucosa indicates their use as early marker for colon cancer. Furthermore, increased EDNRA expression in CRC patients is correlated with increased tumor grade and reduced patient survival (Mahdi et al., 2014). Therefore, endothelin system members are considered as major regulating factors in cancer cell biology and cancer microenvironment and modulation of this gene system in cancer progression is an interesting topic of research.

Our initial approach for mimicking the situation in CC531 colorectal cancer liver metastasis was to induce transient *Edn1* knockdown by siRNA. However, despite using siRNA for different species (human and rat), different CRC cell lines (CC531, LS174T, SW480, SW620), different transfection reagents, and conditions of transfection, there was no significant modulation at mRNA or protein levels (data not shown). The following reasons for this observation can be listed: a) EDN1 is a protein with long intracellular half-life, as was found for bone sialoprotein (Kovacheva et al., 2014). Therefore, the duration of the assay (max. 3 days) was not long enough to detect a meaningful difference at protein level. B) Due to other reasons, which include internal methylation silencing of cytosine residues within promoter areas, the expression of EDN1 was already at its basal limit and could not be lowered further by siRNA techniques. Therefore, and to test this hypothesis, DAC was used to de-methylate cellular DNA of the respective cell lines. In case of a hypermethylation, an increased expression was to be expected in response to DAC exposure.

Patients with metastatic colorectal cancer eventually develop imbalances in promotor methylation, which can result e.g., in reduced expression of tumor suppressor genes and in resistance to chemotherapy. Hypomethylating agents can reverse this resistance mechanism by promotor demethylation and subsequent gene expression reactivation (Lee et al., 2018). In fact, tumor suppressor genes were found re-expressed by DNA methyltransferase enzyme inhibitors (DNMTi) in colorectal cancer cell lines (Bosch et al., 2016). In line with this, DNMT-inhibitors-reversed irinotecan chemotherapy resistance in colorectal cancer models, *in vitro* and *in vivo* (Sharma et al., 2017). Before using DAC as an external modulator, we analyzed DNMT expression from the same set of data. Here, a significantly decreased expression of *Dnmt1* and *Dnmt3b* was recorded early after tumor cell implantation, which indicates a reduced DNA-methylating activity and could explain the increased expression of *EdnrB* and/or *Ece1* mRNA (**Figure 1C**). However, the decreased expression of *Edn1* was probably caused by a different mechanism of action, which might have been activated by the increased levels of *EdnrB* and/or *Ece1* mRNA, as suggested by the short but discernable time lag between the increased expression of *EdnrB* and/or *Ece1* mRNA and the decreased expression of *Edn1*. To better understand this situation, we set up experiments in which human and rat CRC cells were treated with the demethylating agent DAC, which acts through inhibition of DNA methyltransferase enzymes *DNMT1* and *DNMT3* (Ning et al., 2016).

As the use of DAC requires prolonged exposure, we first determined the optimal concentration and time of exposure. In most cancer types, there is inhibition of cell proliferation and motility of cells when exposed to DAC treatment (Li et al., 2015). However, the use of DAC differs from conventional cytostatic antimetabolites. Momparler recommended that DAC doses should be optimized for each type of cancer in order to reach an optimal therapeutic effect as well as minimal side effects (Momparler, 2005a). DAC causes re-expression of silenced genes and may lead to increases or decreases in the expression of other genes. In the panel of CRC cell lines investigated, low µM concentrations (0.5 to 5 µM) of DAC administered daily for 3 days were tolerated sufficiently and caused increased expression of various endothelin system members, which, without this treatment, were present at low mRNA concentrations only.

These data are in line with published data as most experiments performed for profiling the sensitivity to DAC are based on an exposure to DAC for 3 days. In contrast, Stewart et al. relied on 9 days of DAC exposure for assessing changes in chromatin modification. In OVCAR8 cells, a cell line derived from ovarian cancer, the IC50 on day 3 was >33.3 μM, whereas it was 22.2 nM on day 9. In 45 breast, melanoma and ovarian cancer cell lines, there were no marked effects after three days of exposure to DAC, but there were marked reductions in cell viability after 6 and 9 days of treatment, and the sensitivity to DAC markedly increased by 1,000 fold in all cell lines after 9 days of treatment (10 nM < IC50 < 10 μM) (Stewart et al., 2015). The concentrations used in this study were rather in accord with other data, which showed that low DAC doses (below 4 µM) are useful for modulating genes' expression (Egidy et al., 2000; Khimji and Rockey, 2010).

An interesting aspect derived from our cell panel was the observation, that the three metastatic cell lines (CC531, SW620, and LS174T) were on average less sensitive to the anti-proliferative activity of DAC exposure than the four non-metastatic cell lines (SW480, HT29, SW707, and Caco2). If this grouping is related to the antineoplastic activity of DAC, it can be derived that metastasizing cells are more difficult to treat by DAC. Acquired resistance to cancer chemotherapy is due to several factors as epigenetic changes, drug metabolism alteration, enhanced gene amplification and altered DNA repair (Mansoori et al., 2017). For example, HCT116 colorectal cancer cells, resistant to prolonged DAC treatment, showed increased mRNA expression of secreted frizzed related protein 1 and protein expression of cytidine deaminase, while that of deoxycytidine kinase was decreased (Hosokawa et al., 2015).

In addition to these considerations, other mutations could be responsible for the observed resistance to DAC. In fact, all three metastatic CRC cell lines are characterized by a K-ras mutation. In addition, SW620 cells carry a p53 mutation, and LS174T cells harbor a mutated PIK3CA gene, which has implications for the MDM2 expression, as this antagonist of p53 is activated by the PI3K/Akt pathway (Germann et al., 2003; Ahmed et al., 2013). Tumor cells with impaired p53 status are known to show resistance to standard cytotoxic medications, including cisplatin, gemcitabine, and others (Hientz et al., 2017).

The methylation analysis was performed with the lowest concentration (0.25 µM) of this study. Interestingly, in LS174T cells, there was a clear and significant effect in all genes studied except for EDN1, indicating reduced methylation of CpG loci. However, this reduction did not translate into increased gene expression, except for EDN1 and EDN2 genes. This contrasts with the results from SW480 cells, which showed 3.6 fold increased expression of EDN1 RNA, and from SW620 cells, which showed 1.5 fold increased EDN1 RNA levels. Thus, with increasing metastatic properties of CRC cells, there seems to be reduced response to altered methylation levels.

The interpretation of these results could be related to the relative importance of alterations in methylation for induction and progression of cancer. Changes in methylation are early events in carcinogenesis, as has been shown for various types of cancer, including colorectal cancer (Mittag et al., 2006; Liang et al., 2017), breast cancer (Radpour et al., 2011), pancreatic cancer (Hanoun et al., 2010), and lung cancer (Digel and Lubbert, 2005). However, the importance and effect of an altered methylation may decrease during cancer progression. Such a change in importance could result from the cancer cells' capability to deviate from normalcy at both, protein and/or RNA signaling. For example, primary colorectal cancer derived SW480 cells, which were exposed to DAC, exhibited an almost 100fold increase in mRNA expression of *EDN1*, whereas the increased EDN1 expression in metastatic SW620 cells was 50 fold lower than that in SW480 cells, although it was still significant.

This indicates that the tendency of cancer cells for loosing genetic and epigenetic control mechanisms as e.g., the effect caused by promotor methylation, which is active in normal cells, may increase with cancer progression.

So far, the knowledge on the mechanisms by which these changes occur, is still limited. Epigenetic changes in gene expression can be inherited and occur without alterations in a respective DNA sequence (Berger et al., 2009). These changes can result from DNA methylation, histone modifications, and modulation of miRNA levels (Karahoca and Momparler, 2013; Perri et al., 2017). Targeting epigenetic changes by drugs affecting DNA methylation or histone acetylation is an approved tool in cancer therapy (Nie et al., 2014; Ceccacci and Minucci, 2016). It was found that down-regulation of tumor-suppressor genes by hyper-methylation of their promoter is causal for initiation and progression of cancer (Kazanets et al., 2016). Hyper-methylation of promoter regions was found also in genes responsible for regulating cell-to-cell interactions, cell cycle, apoptosis, DNA repair, metabolism of carcinogens, and angiogenesis. Thus, there is an obvious significance of drugs, which affect the methylation status in cancer cells, but their effect may change with the progression of cancer, which can become less or even independent from this mechanism. The results question the success of treating metastatic CRC by methylation inhibitors (Linnekamp et al., 2017). These considerations are paralleled by clinical findings in colorectal cancer patients treated with drug combinations including demethylating agents, in which stable disease was found as best response or even no clinical activity was recorded (Azad et al., 2017; Lee et al., 2018).

In summary, three endothelin system genes (*Edn1*, *Ece1*, and *Ednrb*) were intensively modulated at mRNA level in rat CC531 cells during their liver colonization. An influence from altered methylation was suggested by the concomitant decrease of the two DNA methyltransferases *Dnmt1* and *Dnmt3b*. Changes in methylation accomplished by exposure of CRC cells to the demethylating agent DAC modulated their expression of endothelin system genes at RNA level when using low concentrations, which were continued for 3 days, at minimal cytotoxic side effects. Sensitive human SW480 cells showed an almost 100fold upregulation of *EDN1* mRNA compared to untreated control cells. However, this high mRNA expression in SW480 cells, was not found in metastatic SW620 or LS174T cells. Thus, it is derived that the mechanism exerted by methylation on gene expression can be compromised in metastatic CRC cells.

## Data Availability Statement

The raw data supporting the conclusions of this article will be made available by the authors, without undue reservation, to any qualified researcher.

## Ethics Statement

The animal study was reviewed and approved by Regierungspräsidium Karlsruhe.

## Author Contributions

MM and RG performed the experiments. DA prepared the samples for methylation analysis. MM, RG, and MB analyzed the data and wrote the manuscript. RB, HE, and A-EG gave advice. All authors read and approved the final manuscript.

## Funding

This study was funded by a grant from the Egyptian Mission to MM (CAM 751-FM-06-01).

## Conflict of Interest

The authors declare that the research was conducted in the absence of any commercial or financial relationships that could be construed as a potential conflict of interest.

The reviewer SMK declared a past co-authorship with one of the authors MB to the handling editor.

## Abbreviations

CRC, colorectal cancer; DNMT, DNA methyltransferase; ECE, endothelin-converting enzyme; ECEL, endothelin-converting enzyme-like; ECM, extracellular matrix; EDN, endothelin; EDNRA, endothelin receptor type A; EDNRB, endothelin receptor type B; FCS, fetal calf serum; Luc, luciferase; PBS, phosphate-buffered saline; RFP, red fluorescence protein; RT-PCR, reverse-transcriptase polymerase chain reaction.
