# Supplementary material for: Modulation of the Endothelin System in Colorectal Cancer Liver Metastasis: Influence of Epigenetic Mechanisms?
Source: Front Pharmacol. 2020 Mar 3;11:180. doi: 10.3389/fphar.2020.00180 (PMC7063057; doi:10.3389/fphar.2020.00180)
Supplement: Supplementary file 1 [file DataSheet_1.pdf]

# *Supplementary Material*

## Supplementary Figures

## Figure S1

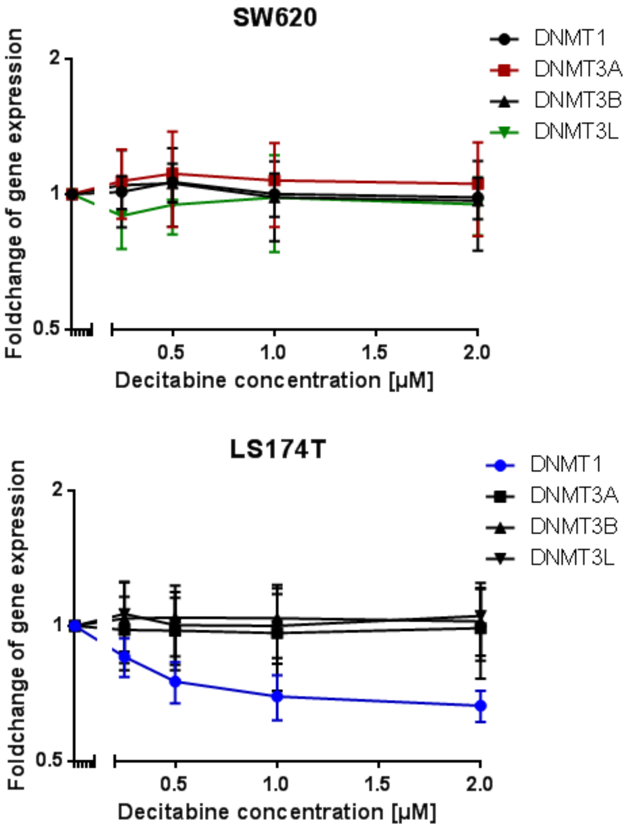


**Figure S1:** Modulation of DNA methyltransferases (*DNMT1*, *DNMT2*, *DNMT3a*, and *DNMT3b*) after exposure to three daily doses of incrementing decitabine concentrations (0.5, 1.0, 1.5, 2.0 µM) in two human (SW620 and LS174T) colorectal cancer cell lines as observed by microarray analysis.
a) Fold-change of gene expression of endothelin system members in SW620 cells.
b) Fold-change of gene expression of endothelin system members in LS174T cells.
The values represent the fold change in gene expression as determined by micro-array in colorectal cancer cells that were exposed to decitabine. The x-axis gives the concentration of decitabine (µM). The y-axis gives the mRNA expression of the studied gene relative to that level found in control cells.

# Supplementary Materials and Methods

## Synthesis of complementary DNA (cDNA)

A reaction mixture was used to synthesize cDNA from isolated RNA. 1000-1500 ng of sample total RNA was mixed with RNase free water, initial heating at 65 °C was done for 5min. Afterwards the following reagents were added per sample: revertAid reverse transcriptase enzyme (2 µl), ribolock RNase inhibitor (1µl), oligo(dt) primer 5´-d(TTTT)-3` (2 µl), dNTP Mix (5 mM each dNTP) (4 µl), 5× buffer (8 µl) and RNAse free water to yield a total volume of 40 µl/sample. Then the mixture was incubated at 42 °C for 60min, followed by a cooling step at 4 °C forever. All reaction materials were supplied from Thermo Scientific.

## DNA band expression by agarose gel

2 µl of cDNA samples were amplified using a Mastercycler® nexus (Eppendorf) by using red taq DNA polymerase (Genaxxon), then the DNA products were separated by electrophoresis in a 2% agarose gel

2 µl cDNA was mixed with a mixture of magnesium chloride ($MgCl_2$) (2 µl, MBI fermentas), dNTPs (1 µl, Thermoscientic), buffer 10× (5µl, Genaxxon), red taq DNA polymerase (0.3 µl, Genaxxon), the selected primer pairs right and left (1µl each), and the remaining amount is double-distilled water (ddH2O) to form total volume of 50µl/sample. Then cDNA was amplified by using the program in table 6 in a Mastercycler® nexus (Eppendorf). Primers used are presented in suppl Table 1. Then the products were analyzed by electrophoresis in a 2% agarose gel.

2% agarose gels were prepared as follows:1X tris-acetate-EDTA (TAE) with 2% agarose were heated at 600 W, then Gel Stain was added (3 µL) after cooling, and the mixture were left to solidify & cast. Samples were run in an electrophoresis chamber at 150 V for 45 min. After electrophoresis, the gel was visualized under ultraviolet light (Gel Doc XR, Bio-Rad, Munich, Germany).

**Table S1:** PCR program used in a Mastercycler® nexus (Eppendorf).

| Steps | Temperature ( °C) | Time | |
|---|---|---|---|
| Initial heating | 95 | 3 min | |
| Denaturation | 95 | 30 sec | Repeated for 35 cycles |
| Annealing | 58 | 1 min | |
| Elongation | 72 | 45 sec | |
| Final elongation | 72 | 5 min | |
| Final hold | 4 | for ever | |

**Table S2:** Endothelin primer sequences and UPL probe numbers (Roche) used for real-time PCR

| | UPL number | Primer Sequence | | Amplicon |
|---|---|---|---|---|
| **Human primers** | | | | |
| endothelin 1 (*EDN1*) NM_001955.4 | 87 | Left | gctcgtccctgatggataaa | 88 nt |
| | | Right | ccatacggaacaacgtgct | |
| endothelin 2 (*EDN2*) NM_001956.3 | 1 | Left | ctatggtctccgtgcctacc | 123 nt |
| | | Right | cttgggcatgagatgaggac | |

| | | | | |
|---|---|---|---|---|
| endothelin 3 (*EDN3*) NM_000114.2 | 22 | Left | acaaagaagaggaagggaaggt | 120 nt |
| | | Right | gggggcaggtagatggag | |
| endothelin converting enzyme 1 (*ECE1*) NM_001397.2 | 54 | Left | actaccgtgcgtgcatga | 102 nt |
| | | Right | agggacctgtgatgttccag | |
| endothelin converting enzyme 2 (*ECE2*) NM_014693.3 | 3 | Left | aacagaactgccaatgagaaagt | 105 nt |
| | | Right | ctgcatctgctccctcgt | |
| endothelin receptor type A (*EDNRA*) NM_001957.3 | 20 | Left | atgtggtcattgatctccctatc | 90 nt |
| | | Right | tgcaaagaaatacgccaaagt | |
| endothelin receptor type B (*EDNRB*) NM_003991.3 | 62 | Left | caatatcttgatcgccagctt | 103 nt |
| | | Right | tccaaatggccagtcctct | |
| **Rat primers** | | | | |
| endothelin 1 (*Edn1*) NM_012548.2 | 63 | Left | caggtccaagcgttgctc | 114 nt |
| | | Right | cctagtccatacgggacgac | |
| endothelin 2 (*Edn2*) NM_012549.2 | 29 | Left | ctggggactctgcctgtg | 76 nt |
| | | Right | ccctggctacttgaggtgac | |
| endothelin 3 (*Edn3*) NM_001077650.1 | 64 | Left | tccttctcgggctcacagt | 112 nt |
| | | Right | accctcttttcctcctggtc | |
| endothelin converting enzyme 1 (*Ece1*) NM_053596.2 | 58 | Left | cctgaacaagacggaaaatga | 121 nt |
| | | Right | caggatctgctgcatctgg | |
| endothelin-converting enzyme 2 (*Ece2*) NM_001002815.2 | 3 | Left | cctaaatagaactgccaatgagaaa | 135 nt |
| | | Right | agttgtatctccagttccagcac | |
| endothelin receptor type A (*Ednra*) NM_012550.2 | 78 | Left | aagtttcctccagccgagac | 143 nt |
| | | Right | ttggtctcacacctttccttc | |
| endothelin receptor type B (Ednrb) NM_017333.1 | 18 | Left | ctgttggcttccccttcac | 107 nt |
| | | Right | tgtagtccaaaaccagcaaaaa | |

**Quantitative real-time PCR (qRt-PCR)**
Real-time PCR was performed using Light Cycler® 480 Probes Master 2X Conc (Roche, Germany), and the human Universal Probe Library kit (Roche, Mannheim, Germany, for probes and primers used see suppl Table 2) was used following the manufacturer's protocol, each well contained (5 µl Master 2× conc, 2.5 µl ddH2O, 2 µl sample DNA, right and left primers (10 µM) 0.2 µl each, universal library probe 0.1 µl, final volume10 µl). Each sample was pipetted in triplicate into 384-well-plates and amplified according to the program in suppl. Table 3. For analysis of the results, the comparative CT method (ΔΔCT Method) was used (Livak and Schmittgen 2001).

**Table S3:** Quantitative real-time PCR program

| | Target (°C ) | Acquisition Mode | Hold (hh:mm:ss) | Ramp rate (C/s) |
|---|---|---|---|---|
| **Pre-incubation** 1 cycle | 95 | - | 00:10:00 | 4.8 |
| **Amplification** 50 cycles, Quantification | 95 | - | 00:00:10 | 4.8 |
| | 59 | single | 00:00:30 | 2.5 |
| | 72 | - | 00:00:01 | 4.8 |

| | | | | |
|---|---|---|---|---|
| **Cooling** 1 cycle | 40 | - | 00:00:30 | 2.5 |

**Western blotting.**

After treatment, cells were harvested, transferred to 1.5-ml micro-centrifuge tubes, washed in PBS and pellets were lysed in RIPA buffer (150 mM sodium chloride, 1.0 % NP-40, 0.5 % sodium deoxycholate, 0.1 % sodium dodecyl sulfate, 50 mM Tris, pH 8.0), and supplemented with complete protease inhibitor cocktail tablets (proteinase inhibitor (25× stock), phosphostop (10× stock), $Na_2VO_3$ (100× stock)) (Roche, Mannheim, Germany). Then the appropriate volume of this solution was added onto samples under continuous agitation by a shaker (30min at -4 °C on ice), then the mixture was centrifuged at 14,000 rpm, 20 min, 4 °C and the supernatant fluid was collected.
Then the protein concentration was determined using the Pierce Protein Assay, for which a relation was used to serial dilutions of standard bovine serum albumin (BSA), and the absorption was read at 450 & 595 nm.
Afterwards each probe containing 40µg sample protein was mixed with 4× LDS sample Buffer NuPAGE (NOVEX Life technology) and 1µl dithiothreitol (DTT; 1M). The probes were mixed by the thermomixer for 5 min at 99 degree at 300 rpm.
Proteins were separated on prefabricated gels (Serva) by electrophoresis at 150 V for 90 minutes in 1× Laemmli buffer. Spectra multicolor broad range protein ladder was used as a size standard.
After that the separated proteins were blotted onto a polyvinylidenfloride (PVDF) western blotting membrane using the A wet-blotting system with transfer buffer (35 V, 60 min).
Then the membrane was blocked with skim milk for 60 min, and the primary antibody was added and left at shaker at 4 °C overnight, afterwards washed with a washing buffer and the appropriate secondary antibody was added for 60 min with continuous shaking at room temperature, and then the membrane was washed again three times with TPBS and then incubated in a mixture of enhanced luminal reagent plus and oxidizing reagent plus for 1 min.
Development of the film was done in the dark in a developing machine (AGFA CURIX 60, Agfa HealthCare). When the membrane was to be reused for another primary antibody, the membrane was stripped by stripping solution for 30 min at 80 °C with continuous shaking, and then the membrane was washed, blocked and re-incubated with the respective primary antibody.

**Knockdown by Small interfering RNA siRNA**

Rat siRNA Edn1-465 (sense: GCGAUCCUUGAAAGACUUA) and human siRNA EDN1-498 (sense: GCUCGUCCCUGAUGGAUAA) (Invitrogen) were used for endothelin 1 knockdown with Xtreme gene or lipofectamine RNAiMAX transfection reagents according to the manufacturer protocols. In short, cells were seeded for 24h in 6 well plates, then the siRNA was added to the cells in combination with one of the transfection reagents.

Two methods for transfection by siRNA were used

- **Xtreme gene transfection reagent protocol**

The colorectal cancer cells were cultured in six well plates in the appropriate number, and then left for 24h before start of transfection. Then, the appropriate amount of siRNA was added to Opti-MEM media to reach a final volume of 100 µl, the appropriate amounts of siRNA are

around 10 µl to 20 µl from siRNA stock of 20 nM to reach final concentration at the cells 100 nM or 200 nM. The transfection reagent Xtreme gene transfection reagent (Roche) was added to opti-media in ratio of two or three in relation to amount of siRNA, according to manufacture protocols, the final volume of solution is 100 µl, then after 5 min incubation in room temperature, the siRNA solution was added to transfection reagent solution, and the mixture was incubated in room temperature for 20 min, then the mixture was added to the cells. Then after 24h, 48h or 72h the cells were collected for assessment of transfection by PCR and western blot.

- **Lipofectamine RNAiMAX reagent protocol**

The colorectal cancer cells were cultivated in 12 well plate in the appropriate number 24h before transfection start, then lipofectamine reagent (Invitrogen) was diluted in Opti-MEM media 6 µl and 100 µl, and siRNA was diluted in opti-Medium 2 µl and 100 µl. After that, the diluted siRNA was added to diluted lipofectamine reagent (1:1 ratio) and incubated for 5 min in room temperature. Then the siRNA-lipid complex was added to cells. Then after 24h, 48h or 72h the cells were collected for assessment of transfection by PCR and western blot.

**Infinium Methylation Assay**

Genome-wide screening of DNA methylation patterns was performed by using the Infinium MethylationEPIC BeadChips (Illumina, San Diego, US), allowing the simultaneous quantitative measurement of the methylation status at 865,918 CpG sites. By combining Infinium I and Infinium II assay chemistry technologies, the BeadChip provides coverage of 99% of RefSeq genes and 96 % of CpG islands (see supplementary file Materials and Methods for more detail).

DNA concentrations were determined using PicoGreen (Molecular Probes Inc., Eugene, USA). The quality of genomic DNA samples was checked by agarose-gel analysis, and samples with an average fragment size > 3kb were selected for methylation analysis. The laboratory work was done in the Genomics and Proteomics Core Facility at the German Cancer Research Center, Heidelberg, Germany (DKFZ).

500 ng genomic DNA from each sample was bisulfite converted using the EZ-96 DNA Methylation Kit (Zymo Research Corporation, Orange, US) according to the manufacturer recommendations. Bisulfite treatment leads to the deamination of non-methylated cytosines to uracils, while methylated cytosines are refractory to the effects of bisulfite and remain cytosine.

Each sample was whole genome amplified and enzymatically fragmented following the instructions in the Illumina Infinium HD Assay Methylation Protocol Guide (genomic DNA). The DNA was applied to Infinium MethylationEPIC BeadChip and hybridization is performed for 16-24h at 48°C. During hybridization, the DNA molecules anneal to locus-specific DNA oligomers linked to individual bead types. One or two probes are used to interrogate CpG locus, depending on the probe design for a particular CpG site.

Allele-specific primer annealing is followed by single-base extension using DNP- and Biotin-labeled ddNTPs. For Infinium I assay design, both bead types (one each for the methylated and unmethylated states) for the same CpG locus incorporate the same type of labeled nucleotide, determined by the base preceding the interrogated “C” in the CpG locus, and therefore are detected in the same color channel. Infinium II uses only one bead type with a unique type of probe allowing detection of both alleles. The methylated and unmethylated signals are generated respectively in the green and the red channels.

After extension, the array is fluorescently stained, scanned, and the intensities at each CpGs were measured. Microarray scanning was done using an iScan array scanner (Illumina). DNA

methylation values, described as beta values, are recorded for each locus in each sample. DNA methylation beta values are continuous variables between 0 and 1, representing the percentage of methylation of a given cytosine corresponding to the ratio of the methylated signal over the sum of the methylated and unmethylated signals. Data analysis was performed with the Illumina's GenomeStudio 2011.1 (Modul M Version 1.9.0).

**Table S4: Methylation pattern of some endothelin system members in decitabine-treated (T) and control (C) human LS174T colorectal cancer cells.**

| Gene[a)] | cgID[b)] | Site[c)] | AVG_Beta (T)[d)] | AVG_Beta (C)[e)] |
|---|---|---|---|---|
| ***Edn1*** | cg08291302 | TSS200;TSS200 | 0,1071455 | 0,1311027 |
| | cg11215644 | 3'UTR;3'UTR | 0,07255715 | 0,08927538 |
| | cg14244360 | TSS200;TSS200 | 0,1253476 | 0,146307 |
| | cg16537245 | TSS1500;TSS1500 | 0,08132226 | 0,1113849 |
| | cg24244437 | TSS1500;TSS1500 | 0,09560417 | 0,1348058 |
| | cg25559625 | TSS1500;TSS1500 | 0,09778348 | 0,1405008 |
| | cg27565517 | Body;Body | 0,0586364 | 0,07348236 |
| | cg20376899 | 1stExon;5'UTR;5'UTR;1stExon | 0,145763 | 0,1775706 |
| ***Edn2*** | cg02634222 | 3'UTR | 0,3708056 | 0,5562898 |
| | cg08460875 | TSS1500;TSS200;TSS200 | 0,2470948 | 0,3064442 |
| | cg14202907 | TSS1500;TSS1500;TSS1500 | 0,3990755 | 0,7638527 |
| | cg14978637 | TSS1500;TSS1500;TSS1500 | 0,5827832 | 0,827497 |
| | cg16736826 | TSS1500;TSS1500 | 0,1373331 | 0,1939932 |
| | cg20367961 | 3'UTR;TSS1500;1stExon;1stExon | 0,5385646 | 0,7219612 |
| | cg20700731 | TSS1500;TSS1500 | 0,1343819 | 0,2146148 |
| | cg21862529 | 3'UTR;3'UTR | 0,4142498 | 0,5216267 |
| | cg27238470 | 3'UTR;TSS1500;1stExon;1stExon;5'UTR | 0,3428136 | 0,4381104 |
| | cg07563342 | 3'UTR;TSS200 | 0,5107244 | 0,7484288 |
| | cg08686022 | 3'UTR;3'UTR | 0,6207902 | 0,9151602 |
| | cg15807154 | TSS1500;TSS200;TSS200 | 0,1840817 | 0,2137354 |
| | cg19874515 | TSS1500;TSS1500 | 0,4230038 | 0,833869 |
| ***Edn3*** | cg00633740 | Body;Body;Body;Body | 0,3918477 | 0,6529219 |
| | cg04048259 | TSS200;TSS200;TSS200;TSS200 | 0,6427182 | 0,9673312 |
| | cg07341690 | TSS1500;TSS1500;TSS1500;TSS1500 | 0,1412242 | 0,2355442 |
| | cg08318212 | TSS200;TSS200;TSS200;TSS200 | 0,4999388 | 0,9439076 |
| | cg09005679 | TSS200;TSS200;TSS200;TSS200 | 0,5764251 | 0,7804127 |
| | cg13919285 | Body;Body;Body;Body | 0,6017595 | 0,8572922 |
| | cg16205854 | TSS200;TSS200;TSS200;TSS200 | 0,604432 | 0,8450123 |
| | cg17146570 | TSS200;TSS200;TSS200;TSS200 | 0,5349287 | 0,951803 |
| | cg17952468 | TSS1500;TSS1500;TSS1500;TSS1500 | 0,2010071 | 0,2560778 |
| | cg20910807 | TSS200;TSS200;TSS200;TSS200 | 0,6396294 | 0,747744 |
| | cg21163415 | Body;Body;Body;Body | 0,2758578 | 0,4018897 |
| | cg01665123 | TSS1500;TSS1500;TSS1500;TSS1500;TSS1500 | 0,2029526 | 0,3093193 |
| | cg04296743 | Body;Body;Body;Body;Body | 0,1016442 | 0,1361438 |
| | cg04477502 | Body;Body;Body;Body;Body | 0,3530108 | 0,5296436 |
| | cg07814082 | 3'UTR;3'UTR;3'UTR;3'UTR;3'UTR | 0,2168905 | 0,3979693 |
| | cg12718049 | Body;Body;Body;Body;Body | 0,1621816 | 0,2371006 |
| | cg14770089 | 3'UTR;3'UTR;3'UTR;3'UTR;3'UTR | 0,3139811 | 0,4902204 |
| | cg14809794 | Body;Body;Body;Body;Body | 0,1010172 | 0,1382381 |
| | cg19905109 | Body;Body;Body;Body;Body | 0,07661048 | 0,1312846 |
| | cg22024589 | Body;Body;Body;Body;Body | 0,08357058 | 0,1145039 |
| | cg19194373 | 5'UTR;1stExon;1stExon;5'UTR;5'UTR;1stExon;1stExon;5'UTR | 0,6344967 | 0,9438744 |
| | cg20671059 | 5'UTR;5'UTR;5'UTR;5'UTR;5'UTR;1stExon;1stExon;1stExon;1stExon;1stExon | 0,6426538 | 0,7966031 |
| | cg23710795 | 5'UTR;5'UTR;5'UTR;5'UTR;5'UTR;1stExon;1stExon;1stExon;1stExon;1stExon | 0,4879484 | 0,5395017 |

| | | | | |
|---|---|---|---|---|
| ***Ednra*** | cg00379467 | TSS200;TSS200;TSS200 | 0,5429557 | 0,7054018 |
| | cg00974629 | TSS200;TSS200;TSS200 | 0,5828776 | 0,8930468 |
| | cg03573792 | Body;3'UTR;3'UTR | 0,3631145 | 0,6595846 |
| | cg03580568 | Body;5'UTR;5'UTR | 0,300923 | 0,3564449 |
| | cg05102394 | Body;5'UTR;5'UTR | 0,5389909 | 0,804934 |
| | cg05618426 | TSS200;TSS200;TSS200 | 0,3216511 | 0,5353364 |
| | cg10112943 | TSS1500;TSS1500;TSS1500 | 0,2969109 | 0,4885649 |
| | cg14948448 | TSS1500;TSS1500;TSS1500 | 0,6130844 | 0,8631078 |
| | cg16264526 | TSS1500;TSS1500;TSS1500 | 0,2218601 | 0,4902192 |
| | cg17073859 | TSS1500;TSS1500;TSS1500 | 0,599831 | 0,8537132 |
| | cg22930773 | Body;Body;Body | 0,2103172 | 0,4094081 |
| | cg23865342 | Body;5'UTR;5'UTR | 0,2647553 | 0,4454416 |
| | cg24789447 | Body;5'UTR;5'UTR | 0,1878639 | 0,245694 |
| | cg01421256 | 5'UTR;Body;Body;Body | 0,510362 | 0,8965383 |
| | cg05857597 | 5'UTR;5'UTR;5'UTR;Body | 0,151121 | 0,3279359 |
| | cg12831879 | 5'UTR;5'UTR;5'UTR;Body | 0,3657451 | 0,746397 |
| | cg13257535 | 5'UTR;Body;Body;Body | 0,2925659 | 0,7008027 |
| | cg14449534 | 5'UTR;5'UTR;5'UTR;Body | 0,5703558 | 0,910007 |
| | cg15500141 | 5'UTR;Body;Body;Body | 0,09199282 | 0,1576776 |
| | cg18057932 | 5'UTR;Body;Body;Body | 0,1178647 | 0,2567794 |
| | cg19207921 | 5'UTR;Body;Body;Body | 0,3392339 | 0,7809858 |
| | cg20402736 | TSS1500;TSS1500;TSS1500;TSS1500 | 0,2271679 | 0,2936738 |
| | cg23718823 | 5'UTR;Body;Body;Body | 0,2246386 | 0,4653105 |
| | cg24782857 | 5'UTR;Body;Body;Body | 0,1664288 | 0,3213757 |
| | cg25705272 | 5'UTR;Body;Body;Body | 0,4723865 | 0,6547812 |
| | cg25984941 | 5'UTR;Body;Body;Body | 0,2075505 | 0,3442599 |
| | cg04045079 | Body;1stExon;5'UTR;1stExon;5'UTR | 0,2998205 | 0,3772426 |
| | cg12009023 | Body;1stExon;5'UTR;1stExon;5'UTR | 0,3712821 | 0,5259702 |
| | cg20557687 | Body;1stExon;5'UTR;1stExon;5'UTR | 0,3106749 | 0,3614736 |
| | cg14986746 | 1stExon;1stExon;1stExon;5'UTR;5'UTR;5'UTR;Body | 0,3352302 | 0,4374267 |
| ***Ednrb*** | cg06106063 | TSS1500 | 0,1563893 | 0,2345513 |
| | cg10042288 | TSS200 | 0,4951259 | 0,696665 |
| | cg10389399 | 5'UTR | 0,2281593 | 0,4736207 |
| | cg10792120 | TSS1500 | 0,1178066 | 0,139707 |
| | cg12321193 | TSS1500 | 0,1144078 | 0,1877395 |
| | cg13432560 | 5'UTR | 0,1990677 | 0,3446122 |
| | cg16203262 | 5'UTR | 0,09030718 | 0,1202725 |
| | cg22154447 | TSS1500 | 0,2621025 | 0,3643319 |
| | cg24588582 | TSS200 | 0,4372807 | 0,8789999 |
| | cg27647038 | 5'UTR | 0,07295255 | 0,1200555 |
| | cg01239435 | TSS1500;5'UTR | 0,1705173 | 0,2125463 |
| | cg08983760 | 5'UTR;1stExon | 0,3546112 | 0,5327781 |
| | cg18003135 | 5'UTR;1stExon | 0,3102905 | 0,4056658 |
| | cg01910869 | TSS1500;5'UTR;TSS1500 | 0,5020744 | 0,5858602 |
| | cg02147695 | TSS1500;5'UTR;TSS1500 | 0,3750089 | 0,604161 |
| | cg03086857 | 1stExon;1stExon;Body | 0,2730309 | 0,3019888 |
| | cg04390523 | 5'UTR;TSS200;TSS200 | 0,5963429 | 0,8213792 |
| | cg06057566 | TSS1500;5'UTR;TSS1500 | 0,5586634 | 0,8603113 |

| | | | | |
|---|---|---|---|---|
| | cg06179060 | 5'UTR;TSS200;TSS200 | 0,1884483 | 0,336491 |
| | cg07035515 | TSS1500;5'UTR;TSS1500 | 0,2089058 | 0,3681335 |
| | cg07495027 | Body;Body;Body | 0,09866967 | 0,1560619 |
| | cg07974719 | TSS1500;5'UTR;TSS1500 | 0,6184321 | 0,9066217 |
| | cg08321129 | TSS1500;5'UTR;TSS1500 | 0,21271 | 0,322584 |
| | cg08634041 | TSS1500;5'UTR;TSS1500 | 0,5380208 | 0,8907503 |
| | cg09152886 | TSS1500;5'UTR;TSS1500 | 0,2147224 | 0,3738428 |
| | cg09786383 | TSS1500;5'UTR;TSS1500 | 0,1716985 | 0,2120599 |
| | cg10016380 | TSS1500;5'UTR;TSS1500 | 0,6209602 | 0,8392283 |
| | cg10764762 | TSS1500;5'UTR;TSS1500 | 0,2681316 | 0,4464264 |
| | cg11074192 | TSS1500;5'UTR;TSS1500 | 0,5480265 | 0,8842228 |
| | cg12120741 | 1stExon;1stExon;Body | 0,5023271 | 0,594822 |
| | cg12602112 | TSS1500;5'UTR;TSS1500 | 0,7159739 | 0,8916239 |
| | cg12847373 | TSS1500;5'UTR;TSS1500 | 0,5023698 | 0,6537069 |
| | cg12935136 | TSS1500;5'UTR;TSS1500 | 0,5983497 | 0,8512115 |
| | cg12983394 | TSS1500;5'UTR;TSS1500 | 0,4464557 | 0,6029329 |
| | cg13434989 | TSS1500;5'UTR;TSS1500 | 0,6253272 | 0,9260656 |
| | cg13818654 | 5'UTR;TSS200;TSS200 | 0,4407227 | 0,5079631 |
| | cg13866767 | Body;3'UTR;3'UTR | 0,3302879 | 0,6709737 |
| | cg15699226 | 1stExon;1stExon;Body | 0,5571828 | 0,9578596 |
| | cg15836660 | 5'UTR;TSS200;TSS200 | 0,404382 | 0,4920833 |
| | cg16739796 | TSS1500;5'UTR;TSS1500 | 0,1499697 | 0,1944652 |
| | cg18032190 | TSS1500;5'UTR;TSS1500 | 0,5793512 | 0,8843021 |
| | cg18568990 | TSS1500;5'UTR;TSS1500 | 0,3548283 | 0,4873351 |
| | cg19650157 | TSS1500;5'UTR;TSS1500 | 0,6906412 | 0,9113404 |
| | cg19742055 | TSS1500;5'UTR;TSS1500 | 0,5685055 | 0,719283 |
| | cg19759502 | TSS1500;5'UTR;TSS1500 | 0,5742196 | 0,8186762 |
| | cg19916212 | TSS1500;5'UTR;TSS1500 | 0,6720599 | 0,8941883 |
| | cg21364111 | TSS1500;5'UTR;TSS1500 | 0,1201939 | 0,1827052 |
| | cg21675115 | TSS1500;5'UTR;TSS1500 | 0,5600346 | 0,8018074 |
| | cg22310279 | TSS1500;5'UTR;TSS1500 | 0,5220866 | 0,821213 |
| | cg22541679 | TSS1500;5'UTR;TSS1500 | 0,3746499 | 0,642967 |
| | cg23326536 | Body;Body;Body | 0,06866998 | 0,08492734 |
| | cg23494140 | TSS1500;5'UTR;TSS1500 | 0,4315396 | 0,6579756 |
| | cg23702615 | TSS1500;5'UTR;TSS1500 | 0,6251598 | 0,9502311 |
| | cg24745738 | TSS1500;5'UTR;TSS1500 | 0,5338176 | 0,851151 |
| | cg24785726 | Body;Body;Body | 0,2138873 | 0,4372701 |
| | cg24942919 | TSS1500;5'UTR;TSS1500 | 0,4123036 | 0,7379285 |
| | cg25717994 | TSS1500;5'UTR;TSS1500 | 0,2159612 | 0,2971753 |
| | cg26022015 | TSS1500;5'UTR;TSS1500 | 0,594691 | 0,8487691 |
| | cg16571983 | 5'UTR;1stExon;5'UTR;1stExon;5'UTR | 0,5824263 | 0,9418846 |
| | cg23316360 | 5'UTR;1stExon;5'UTR;1stExon;5'UTR | 0,5154684 | 0,6827052 |
| ***Ece1*** | cg00054372 | Body;Body;Body;Body | 0,5160899 | 0,8922374 |
| | cg00228796 | Body | 0,4971438 | 0,8751765 |
| | cg00323385 | Body;Body;Body;Body | 0,4523316 | 0,8462206 |
| | cg00344655 | Body;Body;Body;Body | 0,5024834 | 0,8404501 |
| | cg00831171 | Body | 0,4591194 | 0,9005086 |
| | cg00964400 | Body | 0,3552727 | 0,5424841 |

| | | | | |
|---|---|---|---|---|
| | cg01080480 | TSS1500 | 0,5598758 | 0,8840505 |
| | cg01306962 | Body;Body;Body;Body | 0,4136099 | 0,8976192 |
| | cg01383475 | Body;Body;Body;Body | 0,6219062 | 0,9246137 |
| | cg01478827 | Body | 0,4750775 | 0,8693681 |
| | cg01753382 | Body;Body;Body;Body | 0,5447907 | 0,8879702 |
| | cg01758046 | Body;Body;Body;Body | 0,5674443 | 0,9028005 |
| | cg01780990 | Body;Body;Body;Body | 0,5570714 | 0,8008551 |
| | cg01922918 | TSS1500;Body;Body;Body | 0,432688 | 0,7751598 |
| | cg01977520 | Body | 0,5430368 | 0,7648504 |
| | cg02492946 | TSS1500;TSS1500;Body | 0,4034836 | 0,5901228 |
| | cg02582404 | Body | 0,3074535 | 0,3508659 |
| | cg02994246 | Body;Body;Body;Body | 0,4577986 | 0,8621366 |
| | cg03056614 | Body;Body;Body;Body | 0,567679 | 0,8766845 |
| | cg03123991 | Body;Body;Body;Body | 0,5355924 | 0,8959425 |
| | cg03432181 | Body;Body;Body;Body | 0,4442818 | 0,8440905 |
| | cg04114429 | Body;Body;Body;Body | 0,4578052 | 0,8706138 |
| | cg04236915 | Body;Body;Body;Body | 0,4869596 | 0,7770153 |
| | cg04621069 | 3'UTR;3'UTR;3'UTR;3'UTR | 0,5148234 | 0,8569035 |
| | cg05364829 | 1stExon;5'UTR | 0,1250674 | 0,09883196 |
| | cg05370193 | Body;Body;Body;Body | 0,6609569 | 0,9070401 |
| | cg05610701 | Body | 0,4061956 | 0,6938559 |
| | cg05954918 | Body | 0,397916 | 0,6852275 |
| | cg06405255 | Body;Body;Body;Body | 0,6224656 | 0,9286655 |
| | cg06705062 | Body | 0,5913404 | 0,9210311 |
| | cg06819200 | 1stExon;Body;Body | 0,06468166 | 0,08827946 |
| | cg06878749 | Body;Body;Body;Body | 0,5638185 | 0,8984544 |
| | cg07094113 | Body;Body;Body;Body | 0,4114555 | 0,864806 |
| | cg07114751 | Body;Body;Body;Body | 0,5973849 | 0,7964132 |
| | cg07133269 | Body;Body;Body;Body | 0,5847712 | 0,9028306 |
| | cg07157016 | Body | 0,427033 | 0,8564727 |
| | cg07996532 | Body | 0,5330157 | 0,8941814 |
| | cg08146932 | Body;Body;Body;Body | 0,6863397 | 0,8954595 |
| | cg08421373 | Body | 0,4702829 | 0,8894932 |
| | cg08574008 | Body | 0,4864087 | 0,795975 |
| | cg10326339 | Body;Body;Body;Body | 0,5024337 | 0,8619369 |
| | cg10639743 | Body;Body;Body;Body | 0,4887088 | 0,8703957 |
| | cg10699884 | Body | 0,3389252 | 0,448399 |
| | cg10761088 | TSS1500 | 0,0589516 | 0,07407764 |
| | cg10820600 | Body | 0,5625433 | 0,908385 |
| | cg10869378 | Body | 0,461891 | 0,8372829 |
| | cg10984417 | Body;Body;Body;Body | 0,3143419 | 0,4980228 |
| | cg11834658 | Body;Body;Body | 0,6891918 | 0,9075647 |
| | cg12037641 | Body;Body;Body;Body | 0,5285005 | 0,8821381 |
| | cg12216986 | TSS1500;TSS200;Body | 0,03209314 | 0,04916658 |
| | cg12460254 | Body | 0,4492529 | 0,7966434 |
| | cg12771446 | Body | 0,4157157 | 0,8379029 |
| | cg12841412 | Body;Body;Body;Body | 0,580312 | 0,8584121 |
| | cg13086983 | Body | 0,4570371 | 0,865887 |

| | | | | |
|---|---|---|---|---|
| | cg13151223 | Body;Body;Body;Body | 0,5197842 | 0,8891437 |
| | cg13212362 | Body | 0,4495982 | 0,7742246 |
| | cg13447099 | Body;Body;5'UTR;Body;1stExon | 0,5565451 | 0,8638147 |
| | cg13551754 | Body | 0,5132991 | 0,7200916 |
| | cg13562122 | Body;Body;Body;Body | 0,5412646 | 0,8924241 |
| | cg14557415 | Body | 0,3368974 | 0,5458655 |
| | cg14649592 | Body | 0,5126345 | 0,8932124 |
| | cg14783091 | Body;Body;Body;Body | 0,5702599 | 0,8852898 |
| | cg15041744 | TSS200;Body;Body;Body | 0,5637949 | 0,9007236 |
| | cg15462862 | Body | 0,1309985 | 0,1850792 |
| | cg15598754 | Body;Body;Body;Body | 0,6587689 | 0,9484772 |
| | cg15883633 | Body;Body;Body;Body | 0,546877 | 0,8845945 |
| | cg16033503 | Body | 0,5780666 | 0,9294233 |
| | cg16357113 | Body | 0,5754561 | 0,8943019 |
| | cg16385763 | Body;Body;Body;Body | 0,5492553 | 0,9429329 |
| | cg16416158 | Body | 0,4582991 | 0,7328736 |
| | cg16461781 | Body;Body;Body;Body | 0,1397837 | 0,2666322 |
| | cg16514473 | Body | 0,2562492 | 0,4113524 |
| | cg16603916 | Body;Body;Body | 0,6202773 | 0,8185524 |
| | cg16829306 | Body | 0,08215101 | 0,08268329 |
| | cg16910448 | TSS1500;TSS1500;Body | 0,5867798 | 0,9004042 |
| | cg17031944 | Body;Body;Body | 0,5278974 | 0,9112751 |
| | cg17445490 | Body | 0,4861459 | 0,9003378 |
| | cg17446661 | Body | 0,5209528 | 0,8869022 |
| | cg17850498 | Body;Body;Body;Body | 0,4816263 | 0,8463635 |
| | cg18116670 | Body | 0,5158193 | 0,8182775 |
| | cg18574822 | Body;Body;Body;Body | 0,5056469 | 0,8240592 |
| | cg19165640 | Body | 0,5408223 | 0,6947109 |
| | cg19215233 | Body | 0,5577188 | 0,9045973 |
| | cg19273683 | Body | 0,1708786 | 0,2762079 |
| | cg19363242 | Body;Body;Body;Body | 0,6764126 | 0,8584427 |
| | cg19827182 | Body | 0,3922957 | 0,853984 |
| | cg19838586 | Body | 0,4116277 | 0,8486367 |
| | cg19953129 | Body;Body;Body;Body | 0,5592375 | 0,9287599 |
| | cg19987248 | Body;Body;Body;Body | 0,519192 | 0,8675829 |
| | cg20015787 | TSS1500;TSS1500;Body | 0,4710129 | 0,8060876 |
| | cg20554838 | Body;Body;Body;Body | 0,5568978 | 0,8585842 |
| | cg21005416 | TSS1500;Body;Body;Body | 0,2746885 | 0,3444025 |
| | cg21022327 | Body;Body;Body;Body | 0,4989291 | 0,8997188 |
| | cg22040535 | Body;Body;Body;Body | 0,4314674 | 0,8917283 |
| | cg22054164 | TSS1500;TSS1500;Body | 0,5392867 | 0,6966603 |
| | cg22501927 | Body;Body;Body;Body | 0,6117615 | 0,911074 |
| | cg22501992 | Body | 0,5175881 | 0,8991973 |
| | cg22887668 | TSS200;Body;Body;Body | 0,4827111 | 0,7850565 |
| | cg23072086 | TSS1500;Body;Body;Body | 0,4876537 | 0,7971941 |
| | cg23092576 | TSS1500 | 0,06413924 | 0,08903753 |
| | cg23347052 | Body | 0,4975078 | 0,8973305 |
| | cg23470779 | Body;Body;Body;Body | 0,5348771 | 0,9342144 |

| | | | | |
|---|---|---|---|---|
| | cg23764158 | 1stExon;5'UTR;Body;Body;Body | 0,5695956 | 0,9053636 |
| | cg25007705 | Body;Body;Body;Body | 0,3046945 | 0,4447208 |
| | cg25033646 | Body | 0,4682641 | 0,7097588 |
| | cg26348226 | TSS1500;TSS1500;Body | 0,1468259 | 0,2198202 |
| | cg26467313 | Body;Body;Body;Body | 0,4030575 | 0,69133 |
| ***Ece2*** | cg05951558 | Body;TSS1500;TSS1500;TSS1500 | 0,6173308 | 0,8767751 |
| | cg13527631 | Body;Body;Body;Body | 0,5295414 | 0,8296077 |
| | cg22980150 | Body;Body;Body;Body | 0,1115364 | 0,1390877 |
| | cg23872669 | TSS200;Body;TSS200;TSS200 | 0,7095958 | 0,9134879 |
| | cg03835095 | Body;Body | 0,4206189 | 0,8643024 |
| | cg04414966 | TSS200;Body;TSS200;TSS200 | 0,7316712 | 0,8617532 |
| | cg05088549 | Body;Body;Body;Body | 0,2333519 | 0,3095841 |
| | cg07460919 | Body;TSS1500 | 0,3884878 | 0,6252621 |
| | cg07864323 | Body;3'UTR | 0,5289074 | 0,8472652 |
| | cg09970348 | Body;Body | 0,4783616 | 0,6872687 |
| | cg22700017 | TSS200;Body;TSS200;TSS200 | 0,7541537 | 0,8799804 |
| | cg23527621 | Body | 0,5395114 | 0,8535576 |
| | cg23749739 | Body;Body;Body;Body | 0,3414487 | 0,5381204 |
| | cg08770580 | Body;TSS1500 | 0,4936361 | 0,8445451 |
| | cg12232835 | Body;Body | 0,1617173 | 0,2217056 |
| | cg01876619 | Body;Body;Body;Body | 0,7905389 | 0,9514056 |
| | cg14653325 | Body;5'UTR;1stExon | 0,07258382 | 0,08507334 |
| | cg15112081 | Body;TSS1500;TSS1500;TSS1500 | 0,360003 | 0,4987566 |
| | cg01179696 | Body;Body;Body;Body | 0,6599057 | 0,9012217 |
| | cg01189038 | Body;Body | 0,3692151 | 0,4527996 |
| | cg01333080 | Body;TSS1500;TSS1500;TSS1500 | 0,4175735 | 0,7864716 |
| | cg01596834 | Body;5'UTR;1stExon | 0,05489216 | 0,06608124 |
| | cg02791331 | Body;Body;Body;Body | 0,183118 | 0,3064053 |
| | cg03766859 | Body | 0,1144155 | 0,1783217 |
| | cg05118764 | Body;Body | 0,5248614 | 0,9086988 |
| | cg07131336 | Body;Body | 0,4330482 | 0,6673376 |
| | cg08154107 | 1stExon;Body;5'UTR;5'UTR;5'UTR;1stExon;1stExon | 0,6864698 | 0,963538 |
| | cg08832414 | TSS200;Body;TSS200;TSS200 | 0,7140014 | 0,9430322 |
| | cg09405238 | Body;Body;Body;Body | 0,5858308 | 0,5961924 |
| | cg11755372 | Body;TSS1500;TSS1500;TSS1500 | 0,5233252 | 0,8624272 |
| | cg12942257 | Body;Body;Body;Body | 0,4987124 | 0,8522331 |
| | cg19098796 | Body;TSS1500 | 0,08640636 | 0,0973777 |
| | cg19100138 | Body | 0,6091952 | 0,8492848 |
| | cg19141258 | Body;3'UTR | 0,63787 | 0,8882393 |
| | cg22113788 | Body;TSS200 | 0,07880112 | 0,07746631 |
| | cg24976089 | Body | 0,08134003 | 0,1560419 |
| | cg16953297 | ExonBnd;ExonBnd;ExonBnd;ExonBnd;Body;Body;Body;Body | 0,09962696 | 0,2193574 |
| | cg24218885 | Body;Body;Body;Body | 0,4277208 | 0,4676185 |

**[a)]** Gene abbreviation; [b)] Illumina assay probe identification; **[c)]** Site(s) of methylation; [d),e)] An average β-value (AVG_Beta) for each CpG locus ranging from 0 (unmethylated) to 1 (completely methylated) was extracted utilizing the GenomeStudio program (Illumina) for DNA extracted from control (C) and treated (T) LS174 cells.
